# Cellular and Molecular Underpinnings of Neuronal Assembly in the Central Auditory System during Mouse Development

**DOI:** 10.3389/fncir.2017.00018

**Published:** 2017-04-19

**Authors:** Maria Di Bonito, Michèle Studer

**Affiliations:** Université Côte d'Azur, CNRS, Inserm, iBVNice, France

**Keywords:** central auditory system, rhombomeres, DV molecular determinants, *Hox* genes, mouse lineage tracing

## Abstract

During development, the organization of the auditory system into distinct functional subcircuits depends on the spatially and temporally ordered sequence of neuronal specification, differentiation, migration and connectivity. Regional patterning along the antero-posterior axis and neuronal subtype specification along the dorso-ventral axis intersect to determine proper neuronal fate and assembly of rhombomere-specific auditory subcircuits. By taking advantage of the increasing number of transgenic mouse lines, recent studies have expanded the knowledge of developmental mechanisms involved in the formation and refinement of the auditory system. Here, we summarize several findings dealing with the molecular and cellular mechanisms that underlie the assembly of central auditory subcircuits during mouse development, focusing primarily on the rhombomeric and dorso-ventral origin of auditory nuclei and their associated molecular genetic pathways.

## Introduction

Hearing depends on the transmission and transduction of sound stimuli in the inner ear and on processing sensory information in the central nervous system. Proper development and function of both peripheral and central components are necessary for normal sound perception. In this review, we will recapitulate the anatomical, molecular and functional mechanisms involved in the assembly of the central auditory system in the developing mouse. It is known that the unique molecular identity of each rhombomere along the antero-posterior (AP) and the shared genetic pathways of dorso-ventral (DV) domains confer a positional pattern on neuronal progenitors, determining their neuronal subtype identities, migratory pathways, and axonal projections to their proper targets. Several studies have started to correlate early rhombomeric territories and specific DV domains to adult auditory neuronal identity, migration and connectivity patterns (Farago et al., 2006; Louvi et al., 2007; Fujiyama et al., 2009; Maricich et al., 2009; Rose et al., 2009; Di Bonito et al., 2013a; Marrs et al., 2013; Altieri et al., 2015, 2016; Cai et al., 2016). Because of the complexity of the auditory system, the genetic and functional dissection of the different auditory subcircuits have allowed a better comprehension on how neuronal components contribute to distinct pathways with particular functions during development (Maricich et al., 2009; Di Bonito et al., 2013a; Jalabi et al., 2013; Altieri et al., 2014). The availability of *Cre*- and *Flp*-*recombinase* specific mouse lines coupled to reporter lines has become a key tool in labeling genetically defined cell populations and identifying the embryonic origin of auditory hindbrain nuclei. *Conditional knock-in* and *knock-out* transgenic mice have allowed testing the function of individual genes during the construction of the auditory system and the characterization of their pathological role during hearing loss or deafness. Dissecting the anatomical components and functional role of auditory circuits in both normal and pathological conditions contributes to decipher the complexity of sound encoding processes. Moreover, identifying novel genes and molecular pathways specific to distinct rhombomere-specific subcircuits of the developing auditory system is crucial in finding novel players involved in different patterning aspects of hindbrain circuit formation and in the identification of genetic mutations causative of human hearing loss disorders. Below, we will first describe our knowledge about early hindbrain patterning and general anatomy of the auditory system. Then, we will summarize the progress made in the characterization of the rhombomeric and DV origin of auditory hindbrain nuclei and in the identification of genes that along the AP and DV axes are involved in rhombomere-specific formation of distinct functional auditory subcircuits.

## Patterning the hindbrain: a segmentation process crucial for future circuit organization

### Antero-posterior domains: rhombomeres and crypto-rhombomeres

**Figure 1:**
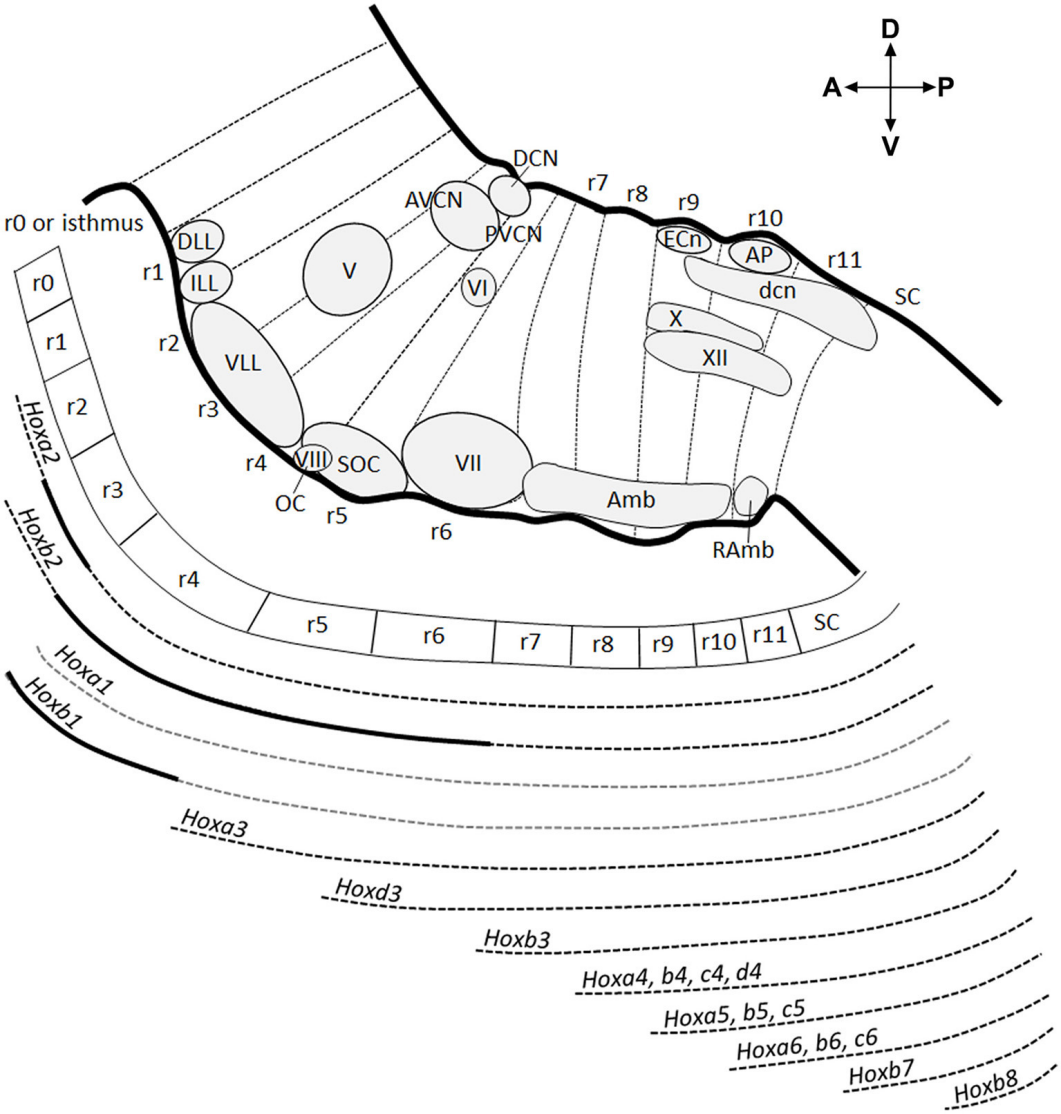
**Antero-posterior anatomical subdivision of the pons and medulla oblongata and corresponding *Hox* gene expression**. Schema showing a sagittal view of the hindbrain subdivided into 12 transverse segments: isthmus and r1 (r0–r1) territories, 5 overt rhombomeres (r2–r6), and 5 crypto-rhombomeres (r7–r11) along the AP axis (above). Below, corresponding *Hox* expression profile correlated to the intersegmentary boundaries and transverse limits of nuclei or intranuclear division. Gray dotted lines refer to *Hoxa1* and *Hoxb1* downregulation during early development. Thick black lines indicate upregulated expression of *Hoxa2* in r3, *Hoxb1* in r4 until E12.5 and *Hoxb2* in r4-r6. Modified from Tomas-Roca et al. (2016).

The hindbrain is an embryonic transient structure, which is subdivided into overt rhombomeres (r) and rhombomere-like hidden segments called pseudo- or crypto-rhombomeres along the AP axis, and which will give rise to the prepontine region including the cerebellum (isthmus or r0, r1, r2), the pons (r3, r4), the retropontine region (r5, r6), and the medulla oblongata (r7–r11) in the adult brain (Puelles, 2013; Puelles et al., 2013; Figure 1). Overt rhombomeres (r2–r6) can be distinguished morphologically in the developing fourth ventricle at early developmental stages, appearing as distinct bulges along the neural tube wall separated by external transverse constrictions and ventricular inter-rhombomeric ridges (Vaage, 1969; Lumsden, 1990). Rhombomeres consists of true segmental compartments displaying typical cellular and molecular characteristics, such as clonal lineage restriction (Fraser et al., 1990; Jimenez-Guri et al., 2010), whereas rhombomeric boundaries have reduced gap-junctional permeability (Martinez et al., 1992), specific cell cycle kinetics (Guthrie et al., 1991) and expression of distinct molecular markers (Heyman et al., 1995). More rostrally, patterning of the isthmus (r0), as well as r1, is not governed by intersegmental boundaries but develop under the influence of gradient signals from the isthmic organizer, forming a morphogenetic field together with the caudal midbrain (Martinez, 2001). The isthmus can be distinguished from r1 only thanks to molecular markers (Aroca and Puelles, 2005; Puelles, 2013). Caudally to r6, the hindbrain appears as an apparently non-segmented region and lacks visible intersegmental boundaries. However, even if transverse constrictions are not distinguishable as in overt rhombomeres, quail-to-chick graft experiments, analysis of *Hox* gene expression domains and cytoarchitectonic landmarks in chicken and mouse medulla oblongata have revealed a hidden transverse organization of the caudal hindbrain, corresponding to a subdivision into five compartments (r7–r11), named crypto-rhombomeres (Cambronero and Puelles, 2000; Marin et al., 2008; Tomas-Roca et al., 2016; Figure 1).

The homeotic *Hox* genes are the earliest genes that delimit rhombomeric boundaries along the AP hindbrain axis. Indeed, the overt inter-rhombomeric boundaries of r1/r2 to r6/r7 correlate with the anterior boundary of Paralog Groups (PG) 1-3 *Hox* genes following the principle of 3′-5′ spatial collinearity (Krumlauf et al., 1993; Parrish et al., 2009; Tumpel et al., 2009). Similarly, the experimentally fate-mapped boundaries of r7–r11 coincide with the rostral expression limits of PG 4-7 *Hox* genes (Marin et al., 2008; Tomas-Roca et al., 2016). Each of these rhombomeres and crypto-rhombomeres, except r1, expresses a particular combination of *Hox* genes, which specify their molecular identity and developmental fate. Both overt and cryptic rhombomere-derived territories persist until adult stages, as demonstrated by quail-chick grafting experiments (Marin and Puelles, 1995; Wingate and Lumsden, 1996; Cambronero and Puelles, 2000) and mouse rhombomere-specific transgenic fate mapping (Farago et al., 2006; Oury et al., 2006; Pasqualetti et al., 2007; Di Bonito et al., 2013a, 2015; Di Meglio et al., 2013; Tomas-Roca et al., 2016). The subdivision of the hindbrain into rhombomeres is highly conserved in vertebrates and is fundamental for the establishment of a complex network of circuits and subcircuits in the adult hindbrain.

Differently from the early rhombomeric organization, the mature hindbrain is organized into longitudinal columns of sensory and motor nuclei ranging from alar to basal and subdivided into discrete segmental units. The columns have a plurisegmental origin, and the molecular boundaries of overt and cryptic rhombomeres correlate topographically with the transverse limits of nuclei or intranuclear subdivisions (Marin and Puelles, 1995; Cambronero and Puelles, 2000; Marin et al., 2008; Tomas-Roca et al., 2016). Moreover, some nuclei display an AP molecular regionalization according to the co-linear differential expression of *Hox* genes (Marin et al., 2008; Tomas-Roca et al., 2016; Figure 1). The original Hox code of each intracolumnar subdivision (jointly with other transcription factors) will then determine the ulterior development of specific neuronal identities and the overall heterogeneity of neuronal populations along the AP axis within sensorimotor columns (see more below).

### Dorso-ventral domains: establishment of neuronal subpopulations

**Figure 2:**
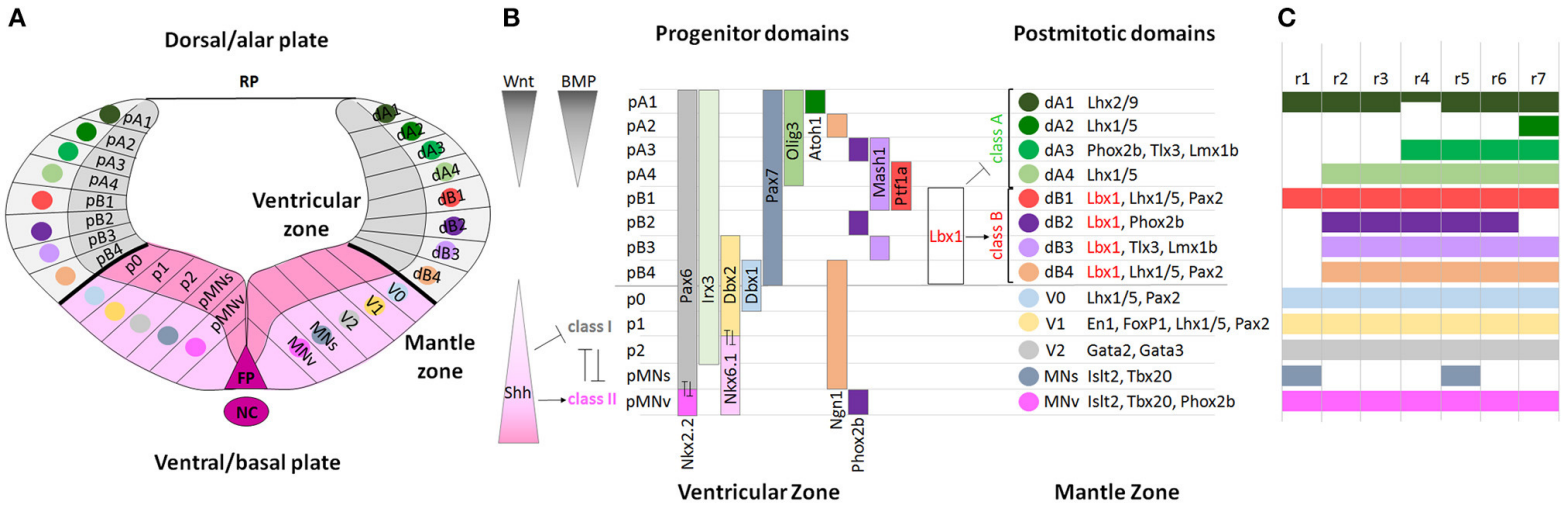
**Molecular specification of dorso-ventral neuronal cell fate in the hindbrain. (A)** Schematic diagram showing the DV organization of a rhombomere from dorsal to ventral: roof plate (RP), dorsal (alar) and ventral (basal) subdomains, floor plate (FP) and notochord (NC). Each domain is formed by progenitors in the ventricular zone and by postmitotic neurons in the mantle zone. **(B)** Two opposite morphogen gradients of Wnt and BMP (dorsalizing signals) produced by the overlying ectoderm and roof plate, and Shh (ventralizing signal) produced by the notochord and floor plate control the expression of bHLH and HD proteins along the DV axis establishing distinct progenitor domains and directing the differentiation of neuronal subtypes. In the alar plate, dA1-dB4 classes of dorsal neurons are subdivided into class A and class B neurons, respectively repressed or activated by *Lbx1* (in red). In the basal plate, HD class I genes are repressed and HD class II genes are activated by Shh signaling, and cross-repressive interactions between class I and class II proteins refine and maintain progenitor domains and determine interneuron (V0-V2), somatic (MNs), and visceral (MNv) motor neuron differentiation. Modified from Takahashi and Osumi (2002), Melton et al. (2004), and Storm et al. (2009). **(C)** Rhombomeric mapping of hindbrain DV domains, modified from Gray (2008). The DV domains extend differently across rhombomeres along the AP axis.

Following the AP rhombomeric divisions, distinct neuronal subtypes arise at stereotyped positions along the DV axis in the developing hindbrain and spinal cord (Figure 2A). The generation of distinct progenitors at defined DV positions in the neural tube depends on two opposite morphogen gradients that act on neuroepithelial cells occupying the ventricular zone of the neural tube: a Sonic hedgehog (Shh) gradient (ventralizing signal) produced by the notochord and the floor plate, and Bone Morphogenetic Protein (BMP) and Wnt gradients (dorsalizing signals) produced by the overlying ectoderm and roof plate (Jessell, 2000; Ribes and Briscoe, 2009; Ulloa and Marti, 2010; Figure 2B). The ventralizing and dorsalizing inductive signals control, in a concentration-dependent manner, the expression of distinct homeodomain (HD) and basic-helix-loop-helix (bHLH) proteins along the DV axis and thus establish progenitor cell domains. Cross-repressive interactions between transcription factors in adjacent domains define sharp boundaries of progenitor domains preventing cells with hybrid identities. Each domain expresses a unique combination of transcription factors that defines the identity of neuronal progenitors, which in turn will give rise to postmitotic neuronal populations expressing specific neuronal-subtype-determining genes (Briscoe et al., 2000; Muller et al., 2002; Takahashi and Osumi, 2002; Lebel et al., 2007; Sieber et al., 2007; Storm et al., 2009; Alaynick et al., 2011). However, the DV domains are differentially represented along the AP axis resulting in some domains, which are reduced or even absent in specific rhombomeres (Figure 2C).

Interactions between the unique molecular identity of each rhombomere and the shared genetic mechanisms of DV patterning will lead to functionally-characteristic alar and basal derivatives of rhombomeres and crypto-rhombomeres, so that rhombomere-specific DV neuronal populations will partially contribute to pluri-neuromeric columnar arrangements (e.g., sensory cochlear, vestibular and trigeminal, as well as motor columns). Inductive signals and specific transcriptional pathways confer a positional identity on neural progenitors, in accordance with their position along the AP and DV axes, and determine the future neuronal fate of distinct neuronal subtypes, specific migratory pathways and axon projections to their corresponding targets (Gaufo et al., 2000, 2004; Pattyn et al., 2003a; Samad et al., 2004; Farago et al., 2006; Jacob et al., 2007; Sieber et al., 2007; Maricich et al., 2009; Storm et al., 2009; Di Bonito et al., 2013a). The regional patterning along the AP axis and neuronal subtype specification along the DV axis intersect and confer distinct rhombomere-specific subcircuits contributing to the complexity of hindbrain sensorimotor systems (Philippidou and Dasen, 2013; Di Bonito et al., 2013b). Long-term fate mapping studies of rhombomeres in chick (Marin and Puelles, 1995; Diaz et al., 1998; Cambronero and Puelles, 2000; Cramer et al., 2000; Marin et al., 2008) and mouse (Farago et al., 2006; Oury et al., 2006; Pasqualetti et al., 2007; Maricich et al., 2009; Rose et al., 2009; Di Bonito et al., 2013a, 2015; Di Meglio et al., 2013; Gray, 2013; Tomas-Roca et al., 2016) have revealed that rhombomeres and crypto-rhombomeres give rise to sensory and motor nuclei of the auditory, vestibular, trigeminal, somatosensory and reticular systems. Single rhombomeres can contribute to nuclei belonging to distinct sensorimotor systems and multiple rhombomeres can contribute to distinct subdivisions of the same nucleus. Hindbrain nuclei relay sensorial information from the periphery to higher brains centers and descending motor information through interconnected neuronal circuits regulating several vital functions. In this review, we will focus on the processes involved in patterning the mouse auditory system and on the associated network of regulatory genes underlying these events.

## General function and organization of the sensorimotor auditory system

**Figure 3:**
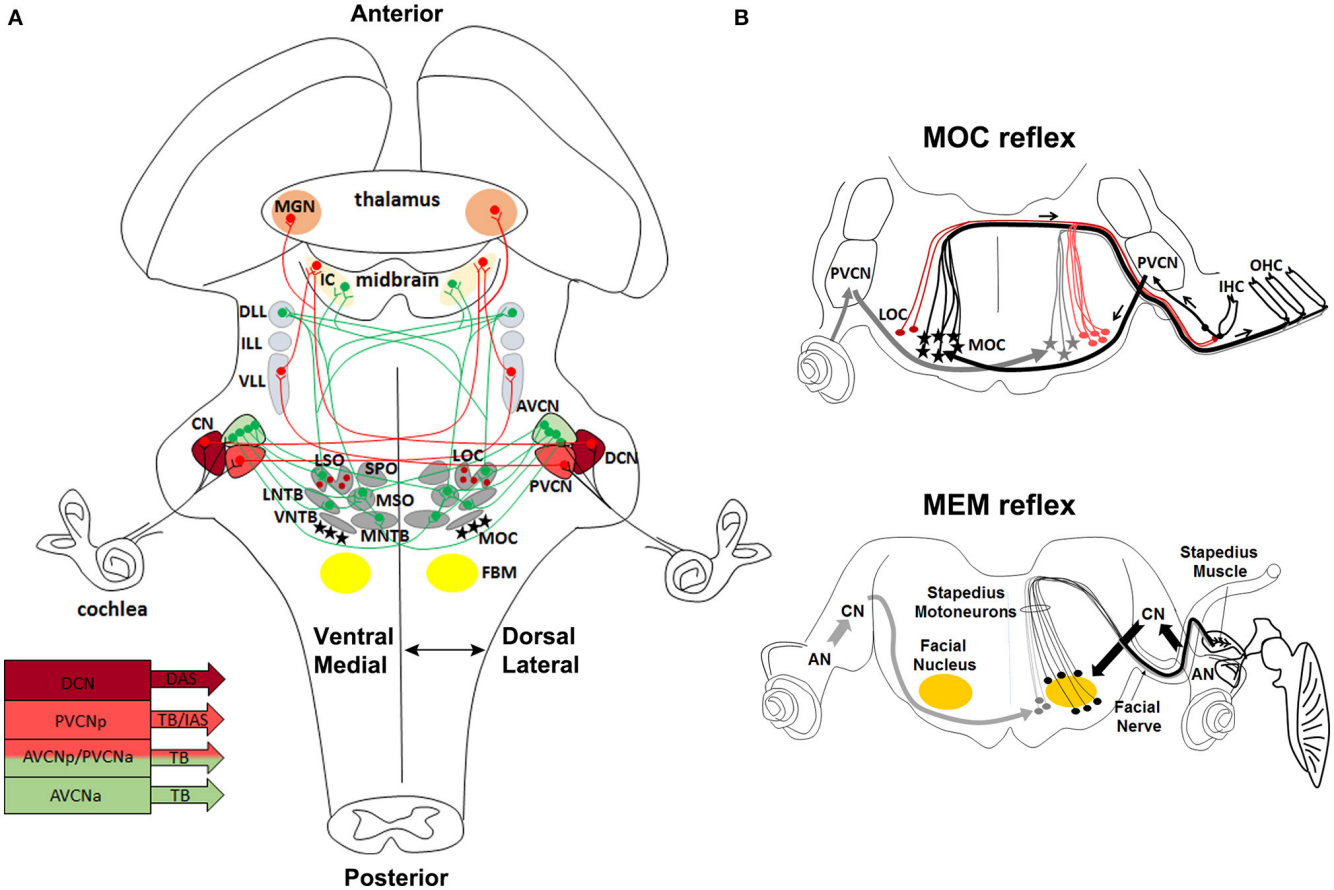
**Anatomical and functional organization of auditory sensorimotor subcircuits. (A)** Anatomical overview of auditory pathways described in the text. Monaural (in red) and binaural (in green) systems receive sound information form one or both ears, respectively. Bottom left, diagram showing three major fiber tracts leaving different parts of the CN: trapezoid body (TB), intermediate acoustic stria (IAS), and dorsal acoustic stria (DAS) modified from Cant and Benson (2003). **(B)** Schema of MOC and MEM reflexes modified from Liberman and Guinan (1998). MOC (contralateral axons in black, and ipsilateral axons in gray) and LOC (red lines) efferent innervation to the cochlea. In the MOC reflex, afferent neurons of the auditory nerve synapse onto cochlear hair cells and project axons centrally to the PVCN. PVCN neurons send axons across the midline to innervate MOC neurons (stars), which mainly project to OHC of the contralateral cochlea and inhibit cochlear responses. The MEM reflex (stapedial reflex pathway) consists of FBM neurons, which are stimulated by VCN interneurons (arrows) and stiffen the ossicular chains by activating the stapedius muscle, thus reducing the sound transmission through the middle ear and offering protection from acoustic overstimulation. AVCNa, anteriorior part of the AVCN; AVCNp, posterior part of the AVCN; PVCNa, anterior part of PVCN; PVCNp, posterior part of PVCN.

Sound consists of alternating compressions and rarefactions propagating through the air. The auricle or pinna captures and conveys sounds to the middle ear, where mechanical energy is transmitted as motions of a chain of three small ossicles: the malleus, the incus and the stapes. Sound-induced mechanical vibrations of the middle ear are transmitted to the cochlea, the sensory end-organ for sound perception in the inner ear, generating in this way cochlear fluid movements. Deflection of the basilar membrane activates the sensory cells that transduce the mechanical energy of sound into electrical signals (Willott, 2001). To receive and elaborate these signals, the cochlea comprises two types of receptors, the inner and outer hair cells. The inner hair cells (IHCs) are the true detectors of auditory stimuli, while the outer hair cells (OHCs) are the amplifiers that enhance low-level sounds by increasing the amplitude and frequency selectivity of basilar membrane vibrations thanks to the “cochlear amplification” process (Ashmore et al., 2010; Guinan, 2013; Goutman et al., 2015). Auditory information is then transferred via the vestibulocochlear (VIIIth) cranial nerve to the brainstem, where auditory nuclei transmit ascending acoustic information, and efferent motor neurons modulate primary afferent responses. In particular, stimuli are first transmitted from IHCs through the primary afferent neurons of the VIIIth nerve to the cochlear nuclear (CN) complex, which is the primary relay station of the central auditory system. The auditory signals travel then from the CN through the main auditory pathway of sound perception consisting of the lateral lemniscus (LL) complex, the inferior colliculus (IC) in the midbrain, the medial geniculate nucleus (MG) in the thalamus, to finally reach the auditory cortex (Willott, 2001; Figure 3A). In a distinct and parallel pathway, excitatory and inhibitory inputs from the CN are first integrated and elaborated by the superior olivary complex (SOC) before being transmitted to higher auditory structures (Grothe et al., 2010; Figure 3A). This pathway is crucial for correct spatial sound localization.

Proper hearing function is also controlled by efferent motor neurons, which modulate afferent sensory auditory stimuli. The olivocochlear neurons (OC) are an important efferent component of the auditory system. In particular, lateral OC (LOC) motor neurons innervate afferent sensory neurons in synaptic contact with the IHCs, modulate cochlear nerve excitability and protect the cochlea from acoustic injury (Simmons, 2002; Darrow et al., 2007), whereas medial OC (MOC) motor neurons are innervated by neurons of the posterior ventral cochlear nucleus (PVCN) (Brown et al., 2003; de Venecia et al., 2005; Darrow et al., 2012) and inhibit vibrating OHCs in the cochlea, thus modulating the sound amplification process through the MOC reflex (Wersinger and Fuchs, 2011) (Figure 3B). Moreover, interactions between cochlear efferent motor neurons and their postsynaptic targets are required for normal development of cochlear function during early postnatal stages (Walsh et al., 1998; Simmons, 2002; Di Bonito et al., 2013a). The middle-ear muscle reflex (MEM) is instead formed by facial and trigeminal branchiomotor neurons (FBM, TBM) that innervate the stapedius and tensor tympani muscles, respectively. The activation of these muscles tense the chain of tympanic ossicles and reduce the amplitude of sound transmission through the middle ear (Liberman and Guinan, 1998; Lee et al., 2006; Figure 3B). Thus, both the MOC and MEM reflexes represent two parallel sound-evoked feedback mechanisms acting, respectively, on the inner and middle ear to modulate incoming acoustic stimuli (Kujawa and Liberman, 1997; Maison et al., 2002; Lee et al., 2006).

## Origins of different components of the central auditory system

**Figure 4:**
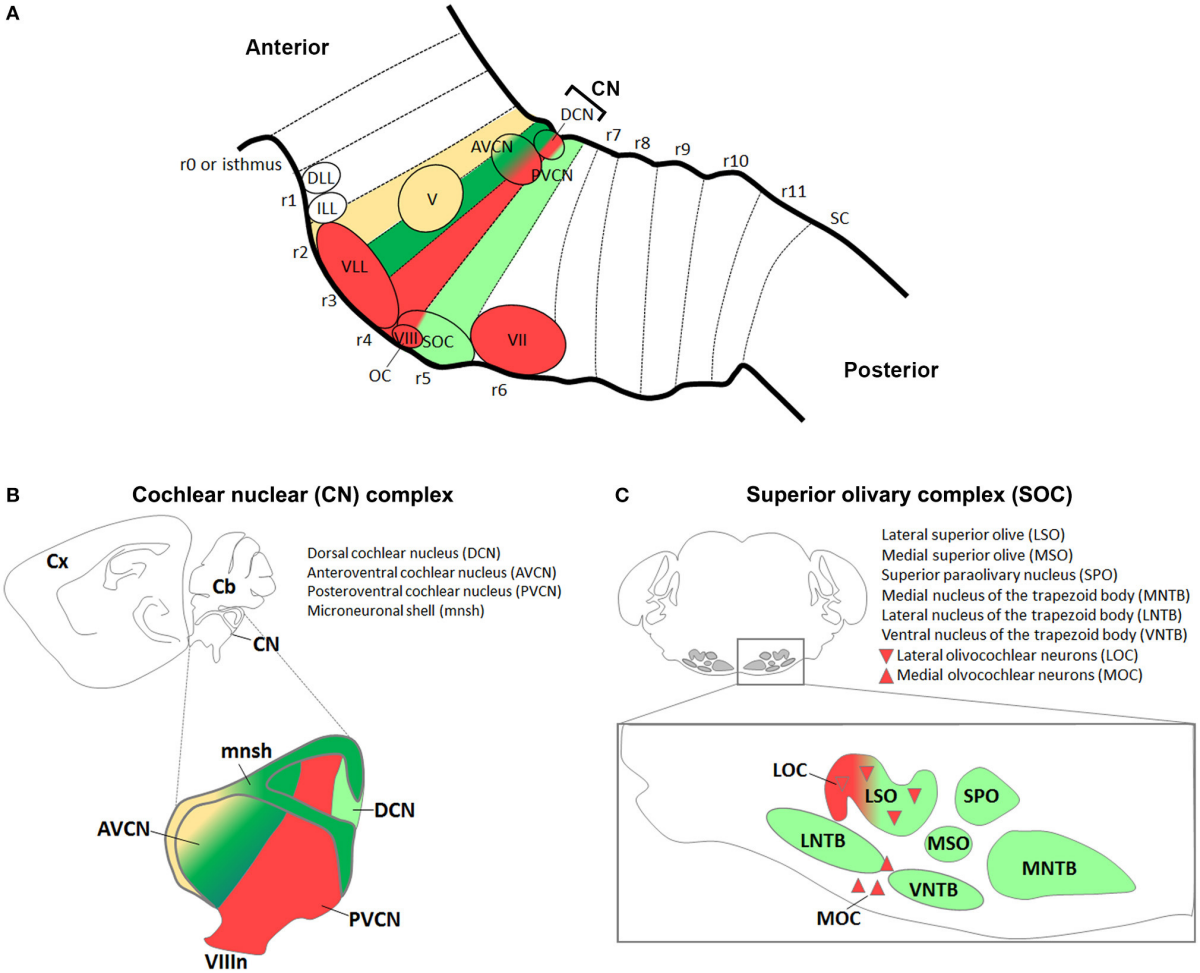
**Antero-posterior origin of auditory nuclei. (A)** Schema showing a sagittal view of the hindbrain in which the color code represents rhombomeric origin of r2- (yellow), r3- (dark green), r4- (red), r5- (light green) derived territories and auditory nuclei. **(B,C)** Schematic representations of the **(B)** cochlear nuclear complex (AVCN, PVCN, DCN, and microneuronal shell), and **(C)** superior olivary complex (LSO, MSO, MNTB, VNTB, LNTB, SPO) and olivocochlear (LOC and MOC) neurons. Colors refer to their rhombomeric origin as described in **(A)**.

**Figure 5:**
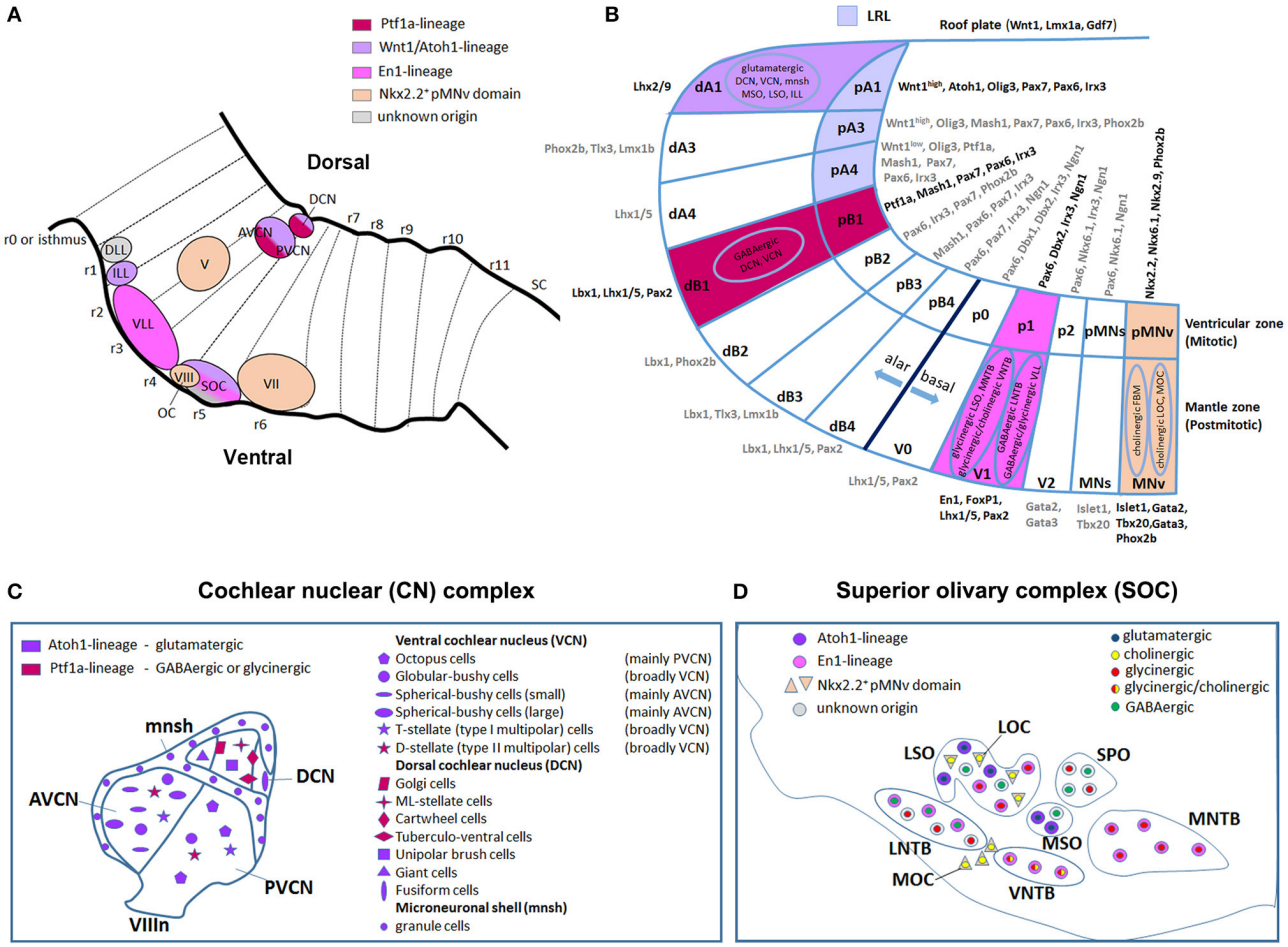
**Dorso-ventral origin of auditory nuclei. (A)** Schema showing a sagittal view of the hindbrain correlating the different auditory nuclei to their DV domains of origin **(B)** by color code. **(B)** Schematic transverse section through the developing hindbrain at r4–r6 levels, showing the DV domain of progenitors and derived neurons and their contribution to auditory nuclei, modified from Nothwang (2016). Note that the dA3 domain is absent in r1–r3, while dA4, dB2, dB3, and dB4 are missing in r1 (Sieber et al., 2007; Gray, 2008). The pMNs domain, dorsal to the pMNv, is only present in r1 and r5 (Takahashi and Osumi, 2002; Guthrie, 2007). **(C,D)** Schematic representation of the DV origin and neurotransmitter phenotype of auditory neuronal populations: **(C)** cochlear nuclear complex [AVCN, PVCN, DCN and microneuronal shell (mnsh)], **(D)** superior olivary complex (LSO, MSO, MNTB, VNTB, LNTB, SPO) and olivocochlear (LOC and MOC) neurons, modified from Fujiyama et al. (2009) and Altieri et al. (2015), respectively. The same color code is maintained from **(A–D)**.

In this section, we will discuss the evidence regarding the origin of sensory and motor auditory nuclei from distinct rhombomeres along the AP axis (Figure 4) and different domains along the DV axis (Figure 5), and summarize the role of transcription factors expressed in DV domains and required in the specification of distinct auditory neuronal subpopulations.

### The cochlear nuclear complex as the primary relay station of the auditory system

The cochlear nuclear (CN) complex is subdivided into ventral (VCN) and dorsal (DCN) cochlear nuclei. While the DCN exhibits a laminar structure that contains the molecular (ML), the fusiform cell (FCL) and the deep (DL) layers, the VCN is further subdivided into anterior (AVCN) and posterior (PVCN) regions (Malmierca and Merchán, 2004; Oertel and Young, 2004; Figures 4A,B, Table 1). The CN is formed by a variety of neuronal subtypes that have different morphological, electrophysiological (e.g., inhibitory or excitatory) and molecular characteristics with unique projection patterns and distinct functional roles during sound processing. The DCN includes Golgi, ML-stellate, cartwheel, tuberculo-ventral, unipolar brush, giant and fusiform cells localized in one or multiple layers, whereas the VCN is formed by octopus, globular bushy, spherical bushy, T-stellate (type I multipolar) and D-stellate (type II multipolar) cells. Neuronal projections leave different parts of the CN via three major tracts, the ventral acoustic stria or trapezoid body (TB), the intermediate and dorsal acoustic stria, and target distinct brainstem nuclei (Cant and Benson, 2003). In particular, axons from the AVCN and the anterior part of PVCN leave the CN via the TB, whereas axons from the posterior part of the PVCN project via the intermediate acoustic stria and those from the DCN via the dorsal acoustic stria (Figure 3A).

**Table 1:** **Summary of antero-posterior and dorso-ventral origin of auditory nuclei**.

**Auditory nuclei**	**AP origin**	**DV origin**	**Geneteic lineage**	**Neurotrasmitter markers**	**Genetic markers (cell type)**	**References**
**COCHLEAR NUCLEUS**						
AVCN	r2-r3 (r4 modestly)	pA1	Atoh1/Wnt1/Wnt3a	VGluT1, VGluT2	Lhx2, MafB Atoh7/Parvalbumin (spherical bushy cells) Atoh7/Calretinin (globular bushy cells)	Wang et al., 2005; Farago et al., 2006; Nichols and Bruce, 2006; Louvi et al., 2007; Yamada et al., 2007; Saul et al., 2008; Fujiyama et al., 2009; Maricich et al., 2009; Rose et al., 2009; Ito et al., 2011; Di Bonito et al., 2013a
		pB1	Ptf1a	Gad67, VIAAT		
PVCN	r4 (r3 modestly)	pA1	Atoh1Wnt1/Wnt3a	VGluT1, VGluT2	Lhx2, MafB Atoh7/Calretinin (globular bushy cells) Calbindin (octopus cells)	Wang et al., 2005; Farago et al., 2006; Nichols and Bruce, 2006; Louvi et al., 2007; Yamada et al., 2007; Fujiyama et al., 2009; Maricich et al., 2009; Rose et al., 2009; Ito et al., 2011; Di Bonito et al., 2013a
		pB1	Ptf1a	Gad67,VIAAT		
DCN	r3-r4-r5	pA1	Atoh1/Wnt1/Wnt3a	VGluT2, VGluT3	Lhx2, Lhx9 Bhlhb5/Pax2/Parvalbumin/CaMKIIα (cartwheel cells) Bhlhb5/Pax6/Tbr2 (unipolar brush cells) Grin2a (giant cells) Grin2b (fusiform cells) Parvalbumin (ML-stellate cells)	Wang et al., 2005; Farago et al., 2006; Nichols and Bruce, 2006; Louvi et al., 2007; Yamada et al., 2007; Fujiyama et al., 2009; Maricich et al., 2009; Rose et al., 2009; Ito et al., 2011; Di Bonito et al., 2013a; Cai et al., 2016
		pB1	Ptf1a	GABA, glycine, Gad67, VIAAT		
mnsh	r2-r3	pA1	Atoh1/Wnt1	VGluT1	Barhl1, Pax6	Wang et al., 2005; Farago et al., 2006; Nichols and Bruce, 2006; Fujiyama et al., 2009; Ito et al., 2011; Di Bonito et al., 2013a
**SUPERIOR OLIVARY COMPLEX**						
LSO	r4/r5	pA1	Atoh1/Wnt1/Wnt3a	VGluT2, Glutamate	MafB	Louvi et al., 2007; Maricich et al., 2009; Rose et al., 2009; Ito et al., 2011; Marrs et al., 2013; Di Bonito et al., 2013a
		p1	En1	Glycine, GlyT2	FoxP1	
		Unknown	Unknown	GABA		
MSO	r5	pA1 Unknown	Atoh1/Wnt1/Wnt3a Unknown	VGluT2, Glutamate GABA	MafB	Louvi et al., 2007; Maricich et al., 2009; Rose et al., 2009; Ito et al., 2011; Marrs et al., 2013
LNTB	r5	p1 Unknown	En1 Unknown	GABA Glycine, GlyT2	FoxP1, Sox2, MafB sparse	Maricich et al., 2009; Marrs et al., 2013; Altieri et al., 2015
VNTB	r5	p1	En1	Glycine, GlyT2, ChAT	FoxP1, Sox2	Maricich et al., 2009; Marrs et al., 2013; Altieri et al., 2015
MNTB	r5	p1	En1	Glycine, GlyT2	FoxP1, Sox2, CaBP, calbindin	Maricich et al., 2009; Ito et al., 2011; Marrs et al., 2013; Altieri et al., 2015
SPO	r5	Unknown Unknown	Unknown Unknown	GABA Glycine, GlyT2	FoxP1, MafB sparse, Gata3	Maricich et al., 2009; Ito et al., 2011; Altieri et al., 2015
**LATERAL LEMNISCUS COMPLEX**						
VLL	r4	p1	En1	Glycine, GlyT2, GAD67, VIAAT	FoxP1, Gata3	Di Bonito et al., 2013a; Altieri et al., 2016
ILL	unknown	pA1	Atoh1/Wnt1/Wnt3a	VGluT2	Lhx2, Lhx9, Barhl2,	Machold and Fishell, 2005; Wang et al., 2005; Louvi et al., 2007; Rose et al., 2009; Ito et al., 2011; Di Bonito et al., 2013a
DLL	Unknown	Unknown	Unknown	VIAAT	Pax7	Ito et al., 2011
**EFFERENT MOTOR NEURONS**						
LOC	r4	pMNv	Nkx2.2/Hoxb1/Phox2b	ChAT	Phox2b, Gata2, Gata3, Tbx20, Islet1	Simon and Lumsden, 1993; Studer et al., 1996; Bruce et al., 1997; Nardelli et al., 1999; Pata et al., 1999; Karis et al., 2001; Tiveron et al., 2003; Di Bonito et al., 2013a
MOC	r4	pMNv	Nkx2.2/Hoxb1/Phox2b	ChAT	Phox2b, Gata2, Gata3, Tbx20, Islet1	Simon and Lumsden, 1993; Studer et al., 1996; Bruce et al., 1997; Nardelli et al., 1999; Pata et al., 1999; Karis et al., 2001; Tiveron et al., 2003; Di Bonito et al., 2013a
FBM	r4	pMNv	Nkx2.2/Hoxb1/Phox2b	ChAT	Phox2b, Tbx20, Islet1	Studer et al., 1998; Auclair et al., 1996; Di Bonito et al., 2013a

*The table summarizes the AP and DV origin of auditory nuclei, their genetic lineage, expression of neurotransmitter and genetic markers, and corresponding references. The genetic markers separated by comma refer to their expression in the entire structure, whereas the ones separated by a slash refer to their expression in specific cell types, listed in parenthesis*.

The cochlear complex originates from distinct regions of the r2–r5 neuroepithelium, which will give rise to the magnocellular cores of AVCN, PVCN and DCN cochlear nuclei, as well as to the cochlear granule cells of the microneuronal shell (mnsh) (Farago et al., 2006; Di Bonito et al., 2013a; Figures 4A,B, Table 1). In particular, r2 gives rise to the AVCN and associated shell, whereas r3/r5 contribute to the AVCN, DCN and shell and only poorly to the PVCN (Farago et al., 2006; Maricich et al., 2009). Differently, r4 largely contributes to the PVCN and the intermediate part crossing dorsoventrally the magnocellular core of DCN, but does not provide any granule cells, and only few r4-derived cells supply the AVCN (Di Bonito et al., 2013a).

### Molecular determinants of the lower rhombic lip pattern different parts of the cochlear nuclear complex

Along the DV axis, the rhombic lip, the dorsal-most neuroepithelial cells of the developing hindbrain, and particularly the lower rhombic lip (LRL) caudal to r1, contributes to all divisions of the cochlear nuclear complex: the magnocellular core regions of the AVCN, PVCN and DCN, as well as granule cells of the microneuronal shell (Wang et al., 2005; Farago et al., 2006; Nichols and Bruce, 2006). The whole rhombic lip expresses *Wnt1*, a member of the Wnt gene family highly implicated in several developmental processes (Awatramani et al., 2003; Landsberg et al., 2005; Nichols and Bruce, 2006). In particular, *Wnt1*^+^ LRL cells from r2 will give rise to the AVCN and associated shell, while r3/r5 *Wnt1*-derived cells contribute to the AVCN, PVCN (poorly), DCN and shell (Farago et al., 2006; Figures 5A,B, Table 1). By using the intersectional and subtractive genetic fate mapping *via* a sophisticated *Flp-FRT* and *Cre-loxP*-based dual lineage-tracing system, Farago and collaborators showed that the magnocellular core of the CN receives contributions from LRL and non-lip progenitors lying ventral to the auditory lip, whereas granule cells derive mainly from LRL (Farago et al., 2006). Interestingly, a graded expression pattern of *Wnt1* along the LRL, high dorsal to low ventral, activates downstream targets in a dose- and spatially-dependent manner defining thus distinct DV progenitor domains (Figures 2B, 5B). In particular, the domain defined as A1, which lies within the *Wnt1*^*high*^ territory, expresses the proneural transcription factor *mouse atonal homolog 1* (*Math1 or Atoh1*) and contributes to distinct auditory nuclei. *Atoh1* is expressed in cycling cells of the ventricular zone (VZ) and in presumed progeny neurons in the immediately lateral mantle zone (MZ) (Landsberg et al., 2005; Figure 5B). In the peripheral auditory system, *Atoh1* is both necessary and sufficient to direct the differentiation and maintenance of cochlear hair cells (Bermingham et al., 1999). Different *Atoh1*^*LacZ*^ and *Atoh1*^*Cre*^ *knock-in* or transgenic mice lines using the *Atoh1* enhancer have allowed to determine the different neuronal populations originating from the *Atoh1*^+^ domain, whereas the availability of *Atoh1*^*null*^ and *conditional* mice has unraveled distinct Atoh1 functions in the central auditory system (Wang et al., 2005; Fujiyama et al., 2009; Maricich et al., 2009; Rose et al., 2009). Fate map studies have shown that cells derived from the r3/r5 *Atoh1*^+^ domain contribute to several glutamatergic neuronal populations of the VCN (mainly to the AVCN) and DCN, as well as to cochlear granule cells (Figures 5A–C). These nuclei are affected in the absence of *Atoh1* demonstrating that *Atoh1* is necessary for the fate determination of CN glutamatergic neurons (Fujiyama et al., 2009).

Granule cells migrate from the LRL into the developing CN via the cochlear extramural stream (CES). Both cochlear granule cells and their precursors express the homeobox gene *Barhl1*, described to act downstream of *Atoh1* (Farago et al., 2006; Rose et al., 2009). Other *Atoh1*-dependent factors, such as *Lhx2* and *Lhx9*, are instead expressed respectively in the DCN/VCN or only in the DCN (Table 1). As expected, expression of all these markers is lost in *Atoh1*^*null*^ hindbrains (Rose et al., 2009), whereas selective deletion of *Atoh1* from r3/r5 results in the loss of the entire AVCN with the exception of a small posterior portion, and in the reduction of both the PVCN and DCN (Maricich et al., 2009). As a consequence, mutant mice have an abnormal auditory brainstem response (ABR) with threshold elevations and absence of the late waveforms generated by the brainstem relay stations. Thus, the auditory nerve properly receives sound information from the inner ear hair cells, but the signal does not propagate beyond the auditory nerve with consequent deafness. The CN dysfunction also causes secondary loss of spiral ganglion (SG) and SOC neurons. The reduction of SG neurons probably accounts for the decreased compound action potentials amplitude found in the *conditional* mutant mice (Maricich et al., 2009). Thus, *Atoh1* represents a true master gene in the development of r3/r5 derived auditory structures.

The *bHLH transcription factor Atoh7* (also known as *Math5, mouse atonal homolog 5*) is expressed in migrating cells of the LRL *Atoh1-*lineage and is also regulated by *Atoh1* (Hufnagel et al., 2007). In the CN, *Atoh7*^+^ cells represent the glutamatergic globular and small spherical bushy neurons distributed with high density in the AVCN and rostral PVCN, and low density in the caudal PVCN (Saul et al., 2008; Table 1). In particular, the spherical bushy cells are predominantly located in the AVCN, while the globular bushy cells are found in the central AVCN and extend into the PVCN. Interestingly, *Atoh7*^*null*^ mice have normal CN dimensions although *Atoh7*^+^ cells are smaller. As a result, ABR tests of the mutant mice revealed decreased II-III and increased III-IV the interpeak latencies. Since the spherical and globular bushy cells and their targets generate peaks II-III and are involved in the integration of binaural auditory information, required for the sound spatial localization, the latency differences due to the dysfunction of *Atoh7*^+^ neurons are consistent with a disruption of binaural processing mechanisms (Saul et al., 2008).

### Other DV domains and their molecular determinants contributing to the auditory cochlear nuclear complex

The *pancreatic transcription factor 1a* (*Ptf1a*) encodes for a basic-helix-loop-helix (*bHLH*) protein expressed in neural progenitors of the A4 and B1 dorsal domains in the mouse hindbrain (Figure 5B). *Ptf1a*-derived neuronal progenies contribute predominantly to the DCN, whereas fewer cells populate the VCN (Yamada et al., 2007; Fujiyama et al., 2009; Figures 5A–C). Particularly, CN excitatory (glutamatergic) neurons arise from the *Atoh1*-expressing LRL, whereas inhibitory (GABAergic or glycinergic) neurons derive from the *Ptf1a*-expressing neuroepithelial domain (Fujiyama et al., 2009; Figure 5B). Thus, the *Ptf1a*^+^ domain supplies GABAergic golgi, ML-stellate cells and glycinergic cartwheel and tuberculo-ventral neurons within the DCN, whereas the glutamatergic unipolar brush, giant and fusiform cells are derived from the *Atoh1*^+^ domain (Figure 5C). Similarly, the *Atoh1*^+^ domain contributes to glutamatergic octopus, globular bushy, spherical bushy and T-stellate cells in the VCN, whereas the *Ptf1a*-lineage gives rise only to glycinergic D-stellate cells. The granule cells in the microneuronal shell are instead glutamatergic *Atoh1*-derivatives. Accordingly, production of inhibitory or excitatory cochlear neurons is severely depleted in *Ptf1a*^*null*^ or *Atoh1*^*null*^ embryos respectively, supporting a specific role for *Ptf1a* and *Atoh1* transcription factors in the development of these populations. However, VCN and DCN neurons with similar electrophysiological profiles (inhibitory or excitatory) derive from the same DV neuroepithelial domains but differentiate into distinct subtypes of inhibitory (e.g., golgi, cartwheel, etc.) or excitatory (e.g., octopus, globular bushy, etc.) neurons. This depends most probably on the integration of the same DV molecular profile with different AP rhombomere-specific genetic pathways. To support this issue, the intersectional genetic fate mapping using subtype- and rhombomere-specific transgenic lines will represent the most appropriate tool to characterize molecular complex mechanisms that generate the vast diversity of CN neurons, and correlate their origin to their neuronal fate and function.

Finally, *Bhlhb5* (also known as *Bhlhe22*) is a neural-specific transcriptional repressor expressed in the DCN during embryonic and postnatal development by the unipolar brush (excitatory) and cartwheel (inhibitory) cells (Cai et al., 2016; Table 1). *Bhlhb5* is required for proper development, ontogeny and survival of DCN neurons, since mice lacking *Bhlhb5* show a dramatically reduced number of unipolar brush and cartwheel cells. The intersectional genetic strategy using a *Bhlhb5Flpo* (an optimized version of the *Flp* recombinase; Kranz et al., 2010), and a *Ptf1aCre* transgenic line was used to specifically label the inhibitory *Bhlhb5*-expressing cartwheel cells. However, the authors show differences in the subpopulations marked by the *Bhlhb5Flpo* and *Bhlhb5Cre* mouse lines. Indeed, while the *Flpo* line identifies cells with high *Bhlhb5* expression levels over an extended period, the *Cre* mouse labels all cells, even if *Bhlhb5* expression is low and/or transient (Cai et al., 2016). Thus, the two lines have distinct patterns of recombination, likely due to the fact that the *Cre*-recombinase is a more active enzyme than the *Flpo*-one in mammalian cells. Finally, a systematic analysis of the transcriptome of individual rhombomeres (r2–r5) has revealed that *Bhlhb5* expression is upregulated in r4 and restricted to the r4 ventral motor neuron domain and to longitudinal stripes in alar r4 (Chambers et al., 2009). Future studies are necessary to determine whether these dorsal domains contribute to *Bhlhb5*-expressing neurons in the DCN in a rhombomere-specific manner.

### The lateral lemniscus complex plays different roles in sound processing

**Figure 6:**
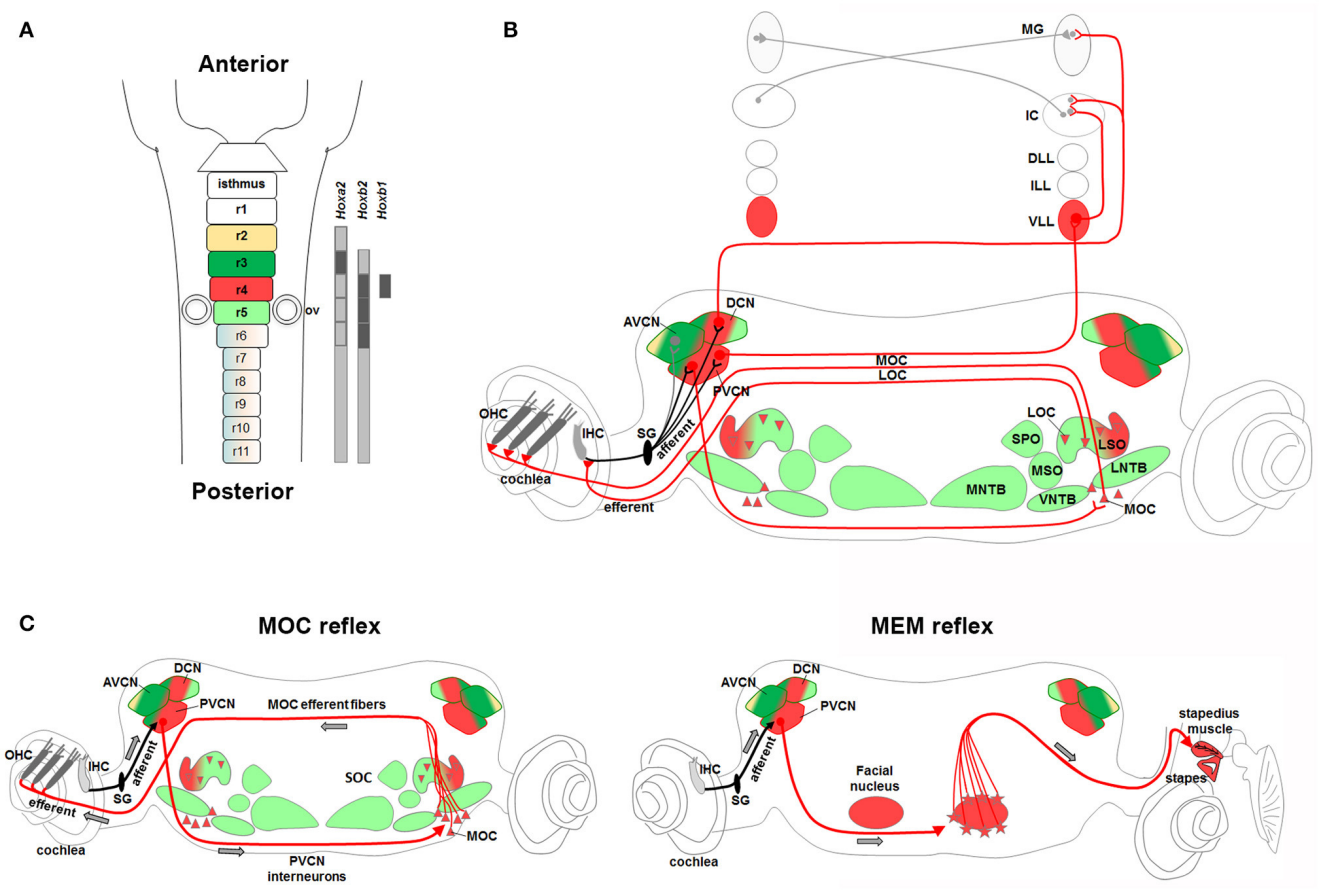
**Rhombomere 4-derived auditory subcircuits**. **(A)** Schema of hindbrain, in which rhombomeres 2–5 are color-coded, and associated *Hox* genes expression. **(B,C)** R4-derived subcircuits involved in the transmission of auditory sounds (CN, VLL), protection from acoustic injury (MOC/MEM reflex and LOC), amplification of low-level sounds (MOC innervation to OHC). **(C)** R4-derived PVCN neurons and motor MOC and FBM neurons contribute to the efferent reflex of MOC and MEM reflex, respectively. Modified from Di Bonito et al. (2013a). *Hoxb1* and *Hoxb2* act primarily upon assembly of r4-derived structures, contributing to the main pathway of sound transmission, as well as in the establishment of sensorimotor reflex circuits important for cochlea protection and amplification processes.

**Figure 7:**
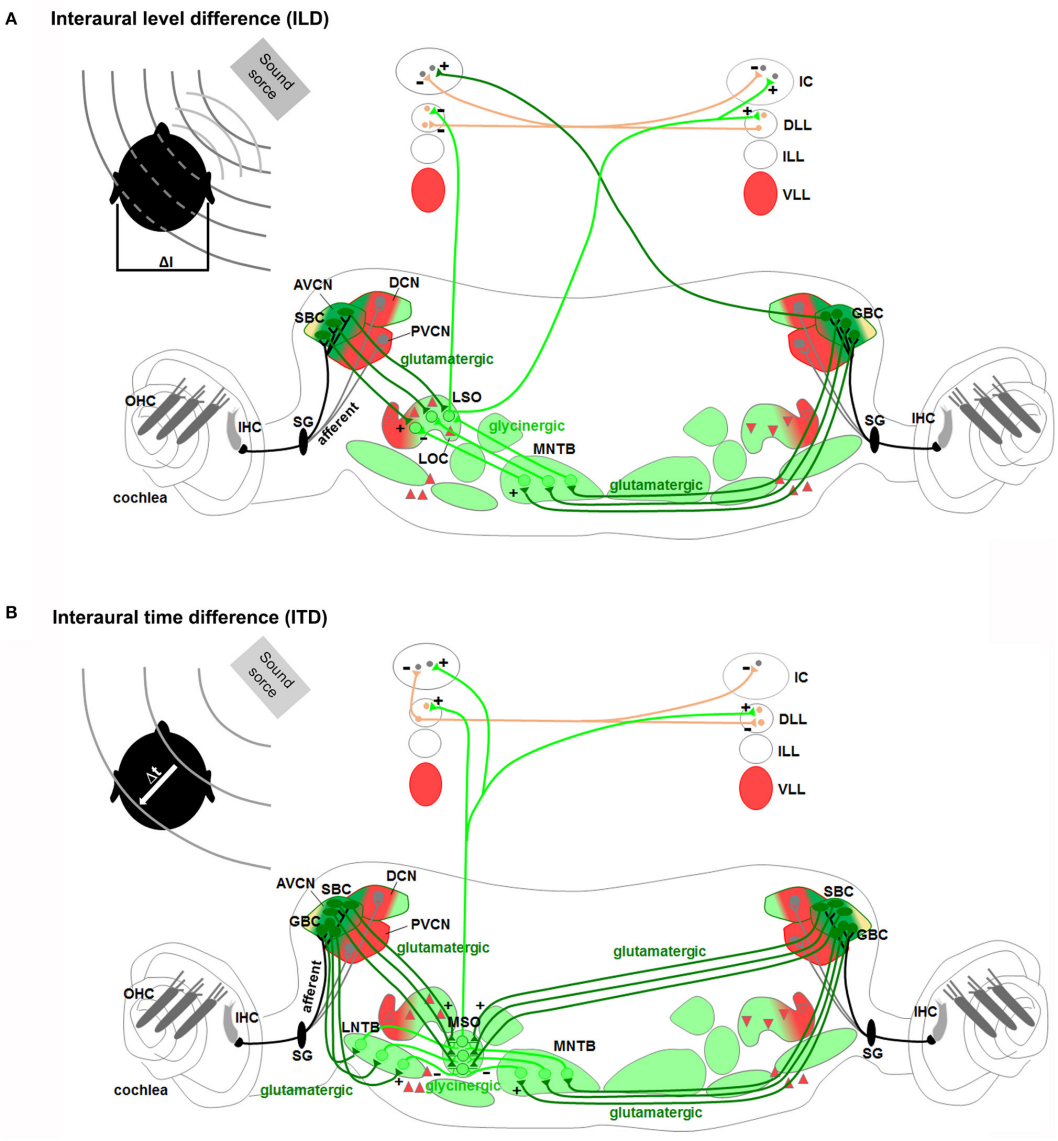
**Rhombomeres 2, 3, and 5-derived auditory subcircuits**. The color code of the auditory nuclei is referred to the schema in Figure 6A. **(A,B)** r2/r3/r5-derived subcircuits involved in the sound localization by **(A)** interaural level difference (ILD) and **(B)** interaural time difference (ITD), modified from Grothe et al. (2010). ILD **(A)** and ITD **(B)** are processed by neurons of r2/r3-derived AVCN, and r5-derived MNTB and LSO or MSO, respectively. *Hoxa2* contributes to AVCN development and connectivity in the sound localization circuit, mainly formed by r2, r3, and r5.

The lateral lemniscus (LL) complex, one of the major stations along the hearing path, is subdivided into ventral (VLL), intermediate (ILL), and dorsal (DLL) nuclei. The VLL receives inputs mainly from the contralateral ear and project to the ipsilateral inferior colliculus (IC) (monaural system), whereas the DLL receives inputs from both ears mainly through connections with the superior olive and projects to both IC (binaural system) (Malmierca and Merchán, 2004; Figures 3A, 6B, 7A,B). The VLL is considered a nucleus primarily involved in processing information concerning the temporal or spectral quality of auditory stimuli (Zhang and Kelly, 2006). The DLL instead is mainly required in the mechanisms of sound localization (Grothe et al., 2010).

Regarding their origins, the VLL derives from basal r4 (Di Bonito et al., 2013a), whereas the rhombomeres that contribute to the ILL and DLL have still to be identified (Figures 4A, 5A,B). The VLL r4-derived neurons migrate rostrally from the basal longitudinal zone along the growing lateral lemniscus tract that ascends through the rostral hindbrain into the inferior colliculus (IC) located in the midbrain (Di Bonito et al., 2013a). The ipsilateral r4-derived VLL neurons and presumably the r4-derived projection neurons of the contralateral CN contribute to form the lateral lemniscus tract that projects to the IC, sends collaterals to the superior colliculus and extends into the medial geniculate nucleus of the thalamus (Di Bonito et al., 2013a; Figure 6B). The r4-derived VLL neurons project to the central nucleus of the IC, whereas r4-derived projection neurons of the CN that contribute to the lateral lemniscus tract, are most likely fusiform cells in the DCN that project to the contralateral IC and medial geniculate nucleus. A recent report has shown that glycinergic and double glycinergic/GABAergic VLL neurons derive from a lineage positive for the homeodomain transcription factor *Engrailed* (*En1*) (Altieri et al., 2016), which is expressed within the V1 postmitotic domain in the spinal cord and hindbrain (Gray, 2008; Alaynick et al., 2011) (Figures 2B, 5A,B). *En1* is necessary for their survival, but not for their generation or positioning, and indeed *En1* deletion causes glycinergic/GABAergic VLL neuronal death during late embryonic and early postnatal development (Altieri et al., 2016). Expression of the Vesicular Glutamate Transporters *VGluT* (*VGluT1, VGluT2*) for glutamatergic neurons, and of the Vesicular Inhibitory Amino Acid Transporter *VIAAT* (also called *VGAT*) for both GABAergic and glycinergic neurons (Ito et al., 2011) confirmed the inhibitory nature of VLL neurons (Di Bonito et al., 2013a; Altieri et al., 2016). Ito et al. (2011) also showed that while DLL neurons are mainly inhibitory with dense clusters of *VIAAT*^+^ neurons and few *VGluT*^+^ neurons, ILL neurons are mainly excitatory with a majority of neurons expressing *VGluT1* and/or *VGluT2*, and a minority expressing *VIAAT*. These new data indicate that the *Atoh1*-derived *VGluT2* glutamatergic component of the LL was inappropriately considered as DLL in previous works (Machold and Fishell, 2005; Louvi et al., 2007; Rose et al., 2009; Di Bonito et al., 2013a), since it most likely represents the ILL. The authors also showed that few *VGluT*^+^ neurons are found in the VLL, and that cells expressing only *VGluT1* form a separate population of small neurons, most likely granule cells, along the surface of the lateral lemniscus. These neurons form a continuous band together with the granule cells of the cochlear nucleus, medial cerebellar peduncle and cerebellum. Finally, cells derived from the *Wnt3a*-lineage also show a similar type of distribution around the VLL, besides contributing to the ILL nucleus (Louvi et al., 2007). More experiments are therefore necessary before understanding the precise origin of ILL and DLL neurons.

### The superior olivary complex as the major component of sound localization

The superior olivary complex (SOC) is a group of brainstem nuclei that act in multiple aspects of hearing and is an important component of the ascending and descending pathways of the auditory system. The nuclei that form the SOC are: the lateral (LSO) and medial (MSO) superior olive, the medial (MNTB), ventral (VNTB), and lateral (LNTB) nuclei of the trapezoid body, the superior paraolivary (SPO) nucleus and the periolivary nuclei (Figure 4C). While r5 gives rise to the majority of SOC nuclei (LSO, MSO, MNTB, VNTB, and LNTB) (Maricich et al., 2009; Marrs et al., 2013), r4 contributes to the *VGluT2*^+^ subpopulation of the LSO and to some periolivary nuclei within the SOC (Di Bonito et al., 2013a and unpublished data; Figure 4C). An r3 additional contribution to the MNTB nucleus has also been suggested (Maricich et al., 2009), whereas nothing is known about the AP origin of the SPO. SOC nuclei have also distinct DV origins: LSO and MSO derive predominantly from the rhombic lip, whereas the MNTB, VNTB, and LNTB nuclei are likely basal plate-derivatives (Maricich et al., 2009; Rose et al., 2009; Marrs et al., 2013; Figures 5B,D, Table 1). In particular, LSO and MSO originate from the *Atoh1*- (Maricich et al., 2009), *Wnt1*- and *Wnt3a*-lineages (Louvi et al., 2007; Marrs et al., 2013), whereas MNTB, VNTB, LNTB mainly derive from the *En1*-lineage (Marrs et al., 2013) that also contributes partially to the LSO. As described above, *Atoh1*^+^ derivatives are glutamatergic (Maricich et al., 2009; Rose et al., 2009), whereas *En1*-derived neurons have multiple neurotransmitter phenotypes: glycinergic, cholinergic and GABAergic (Altieri et al., 2015). Indeed, deletion of *Atoh1* in r3/r5 causes glutamatergic neuronal loss in LSO and MSO and secondary reduction of neurons in the MNTB, VNTB, and LNTB (Maricich et al., 2009). *Conditional* deletion of *En1* in r3/r5 instead leads to the absence of MNTB and VNTB nuclei, which are the two nuclei entirely derived from the *En1*-lineage (Jalabi et al., 2013). A further report has also shown that LSO and MNTB glycinergic neurons, VNTB glicinergic/cholinergic neurons and the GABAergic component of the LNTB derive from *En1* and are lost in r3/r5 *En1 conditional* mutant mice, as a consequence of neuronal death (Altieri et al., 2015).

### Brainstem subcircuits involved in sound localization

Proper spatial information on the horizontal (azimuth) and vertical (elevation) directions and distance of the sound source from the listener is required for sound localization. Sound localization in vertical plane and in horizontal front/back position is a monaural pathway, which uses modifications in the sound spectra produced by the interactions of sound with the asymmetric external ear (Grothe et al., 2010). The DCN is particularly specialized in the first processing of spectral localization cues and in conveying these signals to the IC (Cant and Benson, 2003; Oertel and Young, 2004; Grothe et al., 2010). Mechanisms of localization for incoming sound source in the horizontal plane (excluding front/back localization) are instead based on two different binaural pathways processing differences in the interaural time (ITDs) and interaural level or intensity (ILDs or IIDs) between sounds reaching the two ears (Kandler and Gillespie, 2005; Grothe et al., 2010; Figures 7A,B). The ITDs depend on the different distance that a sound wave must travel to reach the near and far ears, which will determine the difference in the arrival time. ILDs depend on the shadowing effect of the head on sound that reaches the ear further from the source, which creates differences in the sound level at the two ears. The binaural ILDs and ITDs for the azimuthal sound localization are first processed by the LSO and MSO, respectively (Kandler and Gillespie, 2005; Grothe et al., 2010).

LSO neurons encode ILDs by integrating excitatory inputs from the ipsilateral ear with inhibitory inputs from the contralateral ear so that neurons are completely inhibited when sound at the contralateral ear is more intense, and highly responsive when sound at the ipsilateral ear is more intense (Figure 7A). In particular, LSO principal neurons receive excitatory glutamatergic inputs from the small spherical bushy cells of the ipsilateral AVCN and inhibitory glycinergic inputs from the MNTB, which in turn is activated by excitatory glutamatergic inputs from the globular bushy cells of the contralateral AVCN. LSO neurons send excitatory projections to the contralateral DLL and IC and inhibitory ones to the ipsilateral DLL. In the IC, ILDs are created by the convergence of monaural contralateral excitatory inputs from the AVCN and binaural inhibitory inputs from the DLL. MSO principal cells with bipolar morphology encode ITDs by integrating bilateral excitatory inputs from both cochlear nuclei and bilateral inhibitory inputs through the LNTB and the MNTB (Figure 7B). MSO neurons receive tonotopic binaural excitatory glutamatergic inputs from large spherical bushy cells in both AVCNs with ipsilateral axons synapsing on the lateral dendrites and contralateral axons on the medial ones. In addition, MSO neurons also receive binaural inhibitory glycinergic inputs to their soma from the ipsilateral LNTB and MNTB, which in turn are stimulated by excitatory glutamatergic inputs of ipsilateral and contralateral AVCN globular bushy cells, respectively. Finally, MSO neurons send excitatory projections to the DLL and IC.

Components of the spatial sound localization pathways have different rhombomeric origins and derive from specific lineages. *Atoh1*-derived *Atoh7*^+^ spherical and globular bushy cells in the AVCN originate from r2/r3, while *En1*-lineage glycinergic neurons of the MNTB derive from r5, supporting the main contribution of these rhombomeres to the sound localization process. However, the ability to localize sounds and glycinergic innervation of the SOC persist after genetic deletion of the MNTB, observed upon loss of En1 function in r3/5 (Jalabi et al., 2013). In these mice, there is a complete lack of MNTB and VNTB neurons, whereas LNTB neurons and the functional glycinergic innervation of the LSO and SPO are relatively preserved. This could be due either to the developmental compensation by other regions of the central auditory system, or to parallel inhibitory innervation, normally existent but obscured in wild type animals in which glycinergic MNTB innervation dominates. Finally, it suggests that there are other sources of glycinergic innervation and other auditory structures that might contribute to the sound localization process (Jalabi et al., 2013; Altieri et al., 2014).

### Efferent motor neurons contribute to the MOC and MEM reflexes

Hair cells in the inner ear receive efferent innervation through the VIIIth cranial nerve from motor neurons located in the hindbrain (Figure 3B). The inner ear efferent (IEE, also known as contralateral vestibuloacoustic or CVA) neurons originate from the progenitor domain of visceral motor neurons (pMNv) in ventral (basal) r4 (Simon and Lumsden, 1993; Bruce et al., 1997), which also gives rise to another efferent neuronal populations, the facial branchiomotor (FBM) neurons of the VIIth cranial nerve (Auclair et al., 1996) (Figures 5A,B). R4-derived IEE neurons can be subdivided into vestibular (VEN) and cochlear (CEN) efferent nuclei, according to their targets. CEN form two groups of olivocochlear neurons (OC): the lateral (LOC) and medial (MOC) olivocochlear efferent neurons, located within the LSO and in the medioventral portion of the SOC as spread cells, respectively (Brown and Levine, 2008; Figures 4C, 5D). LOC neurons project mainly to the ipsilateral cochlea and terminate on afferent sensory dendrites contacting IHCs, modulate cochlear nerve excitability and protect the cochlea from acoustic injury (Kujawa and Liberman, 1997; Darrow et al., 2007; Figure 3B). MOC neurons instead project mainly to the contralateral cochlea innervating directly OHCs and modulate the cochlear amplification process by inhibiting OHCs motility through the MOC reflex (Ashmore et al., 2010; Figure 3B). The MOC reflex is a three-neuron pathway (Guinan, 2006): (i) sound excites auditory afferent neurons of the auditory nerve (SG neurons) that synapse on cochlear hair cells in the periphery and innervate reflex interneurons in the PVCN; (ii) these PVCN interneurons are planar multipolar cells (corresponding to the T-stellate or type I multipolar cells) (de Venecia et al., 2005; Darrow et al., 2012) and send axons across the midline to innervate MOC neurons; (iii) MOC neurons project to cochlear OHCs and inhibit cochlear amplification reducing basilar membrane responses to low-level sounds (Figures 3B, 6C). Another reflex which protects the ear from overstimulation is the middle ear muscle (MEM) reflex, formed by facial (FBM) and trigeminal (TBM) branchiomotor neurons that innervate muscles of the stapedius attached to the head of the stapes and tensor tympani inserted onto the malleus, respectively (Liberman and Guinan, 1998; Lee et al., 2006). Thus, by stiffening the ossicular chain and reducing the amplitude of sound transmission through the middle ear, the MEM reflex offers protection from acoustic overstimulation. In particular, the stapedial reflex pathway begins with the excitation of the auditory nerve that in turn acts on MEM reflex interneurons in the VCN (Figure 3B, 6C). Output from the VCN leads to the excitation of r4-derived FBM motor neurons and consequent contraction of the stapedius muscles attached to the stapes. The stapedius muscles and stapes are both derivatives of the 2nd pharyngeal arch neural crest cells originating from r4 (Anthwal and Thompson, 2016).

Hence, r4 represents a crucial rhombomere in the development of the central auditory system (Figure 6). While r4-derived CN and VLL contribute to the ascending sound transmission pathway, ventral r4-derived efferent neurons (MOC, LOC, and FBM) are involved in the processes of sound amplification and protection from acoustic injury. R4-derived cochlear sensory neurons (PVCN interneurons) form jointly with r4 basal plate-derived motor neurons (FBM and MOC) two distinct auditory sensorimotor feedback subcircuits required in modulating incoming acoustic stimuli at the level of the middle and inner ear (Di Bonito et al., 2013a). On the contrary, sound localization pathways are mainly derived from r2, r3, and r5 (Figure 7). Therefore, structurally and functionally interconnected components of the auditory system are specified in a rhombomere-specific manner and different rhombomeres give rise to distinct subcircuits with diverse functions.

### Considerations in the use and interpretation of rhombomeric-specific transgenic lines

The rhombomere-specific *Cre* transgenic mice have represented a powerful tool for identifying and characterizing the origin of the different components of the central auditory system. However, some aspects need to be taken into account for a correct interpretation of published data. The *Egr2Cre* (*r3/r5Cre*) (Voiculescu et al., 2000) has been regularly used to fate map or alter r3- and r5-derived neuronal populations (Wang et al., 2005; Maricich et al., 2009; Rose et al., 2009), because neither r3- or r5-specific *Cre* lines were available, or anyway used in these studies. However, it is important to consider that there is no way to distinguish r3- from r5-specific derivatives using this transgenic line. Thus, to avoid misleading interpretations, it is important to take into account that the mapped populations not necessarily derive from both rhombomeres but could arise only from r3 or r5. In addition, it can be risky to presume the rhombomeric origin of a specific cell population just by subtracting it from the previously mapped one. For example, differently from what was initially hypothesized (Farago et al., 2006), r4 does not contribute to the cochlear granule cells of the microneuronal shell (Di Bonito et al., 2013a), which instead origin from r2 and r3, as confirmed by ectopic production of granule cells in *Hoxb1* mutant mice in which r4 acquires an r3 identity (Farago et al., 2006; Di Bonito et al., 2013a). Thus, it seems unlikely that they also originate from r5, as indirectly hypothesized using the *Egr2Cre* line. Moreover, the MNTB might only derive from r5 since it is located in r5 (Marrs et al., 2013), and no migration from r3 has ever been described. On the other hand, the r4-derived DCN region occupies only an intermediate sector within the magnocellular core of the DCN (Di Bonito et al., 2013a) between two small portions; thus, while the posterior DCN derives from r5, as already reported (Farago et al., 2006; Maricich et al., 2009), the anterior part most likely originates from r3, not previously taken into account in the fate map analysis of the *Egr2Cre* line (Figure 4B). In this scenario, the AVCN and granule cells derive from r2 and r3 with only few cells of r4 origin in the AVCN, whereas the PVCN is mainly an r4-derived structure with a modest contribution of r3, and the DCN receives a multi-rhombomeric contribution from r3, r4 and r5 (Figures 4A–C, Table 1). Finally, before the publication of a very specific *r4-Cre* transgenic line (called *b1-r4Cre*) (Di Bonito et al., 2013a), a *Hoxb1Cre* knock-in line was used to fate map r4-derivatives, but since *Hoxb1* is also expressed caudal to r4 at early stages (Murphy et al., 1989; Arenkiel et al., 2003), studies using this line cannot exclude a contribution of posterior rhombomeres (Maricich et al., 2009; Marrs et al., 2013).

## Role of *Hox* genes in the assembly of central auditory subcircuits

As previously mentioned, *Hox* genes are homeotic genes required in conferring rostrocaudal identity of rhombomeres along the AP axis during hindbrain segmentation, and in specifying distinct neuronal populations along the DV axis during neurogenesis (Tumpel et al., 2009; Philippidou and Dasen, 2013; Di Bonito et al., 2013b). In the hindbrain, PG 1-7 *Hox* genes display expression patterns with defined rostral boundaries according to the 3′ to 5′ collinearity rule, whereby 3′-located genes will have a more rostral expression boundary than genes located more 5′ in the same cluster (Duboule and Dolle, 1989; Graham et al., 1989; Wilkinson et al., 1989; Lumsden and Krumlauf, 1996; Marin et al., 2008; Tomas-Roca et al., 2016). Except r1, which is devoid of *Hox* expression, each rhombomere expresses a distinct Hox combinatorial code that specifies the identity and patterning program of each hindbrain segment (Krumlauf, 1993b; Maconochie et al., 1996; Tumpel et al., 2009).

*Hox* genes become progressively restricted along the DV axis into specific progenitor domains during neurogenesis contributing hence to proper specification of distinct neuronal subpopulations (Davenne et al., 1999; Gaufo et al., 2000, 2003, 2004; Pattyn et al., 2003a; Samad et al., 2004; Jacob et al., 2007). The early segmental *Hox* expression patterns are often maintained through later stages in subsets of rhombomere-derived progenitors, postmitotic and projection neurons, which will contribute to specific developing brainstem nuclei. Several studies have demonstrated that *Hox* genes play important roles in several aspects of sensorimotor circuit development and assembly regulating neuronal fate, stereotypic neuronal migration and axon pathfinding patterns in a rhombomere-specific manner (Oury et al., 2006; Geisen et al., 2008; Chen et al., 2012; Di Bonito et al., 2013a, 2015; Di Meglio et al., 2013). Thus, rhombomere-specific (AP) and alar- to basal-restricted (DV) pools of neurons contribute to distinct functional pathways and circuits through the maintenance of differential combinations of *Hox* genes that, in turn, will continuously refine regional identity within the multi-segmental neuronal columns. This implies that single rhombomeres and their specific *Hox* gene combinations are involved in generating different components within distinct sensorimotor systems, contributing in this way to build the complex network of circuits and functional topographic connectivity in the mature hindbrain (Narita and Rijli, 2009; Di Bonito et al., 2013b; Philippidou and Dasen, 2013).

To this regard, mutations in human *HOX* genes have been correlated with hearing impairments (Quinonez and Innis, 2014; Willaredt et al., 2014). Homozygous mutations of *HOXA1* are associated with the autosomal recessive Athabascan Brainstem Dysgenesis Syndrome (ABDS) and the Bosley-Salih-Alorainy Syndrome (BSAS) characterized by sensorineural deafness due to the absence or aplasia of the cochlea (Tischfield et al., 2005; Bosley et al., 2008). Furthermore, homozygous missense mutation in *HOXB1* has been linked to sensorineural hearing loss in patients with Moebius syndrome (Webb et al., 2012). ABR tests have revealed bilateral mild to moderate high-frequency hearing loss with normal waveform latencies, whereas no distortion product otoacustic emission (DPOAE) was recorded in the cochlea, indicating abnormal cochlear OHC function. Finally, heterozygous nonsense (haploinsufficiency) or homozygous missense (reduced DNA binding affinity) mutations in *HOXA2* causes a dominant or recessive bilateral microtia respectively, a rare congenital malformation of the external ear, as well as hearing loss (Alasti et al., 2008; Brown et al., 2013).

Can mouse genetics help us in better dissecting patients' hearing abnormalities? The PG1 *Hoxa1* and *Hoxb1* genes are activated in the mouse hindbrain by retinoic acid and expressed with an anterior border at the presumptive r3/r4 boundary at early embryological stage before the formation of definitive rhombomeres (Marshall et al., 1994; Dupe et al., 1997; Studer et al., 1998; Figure 1). *Hoxa1* transactivates *Hoxb1* expression and both synergize in establishing *Hoxb1* expression in r4 by binding to an r4 enhancer located in the 5′ promotor region (Studer et al., 1998). Then, *Hoxb1* is selectively maintained in r4 by an autoregulatory loop which is repressed in adjacent rhombomeres (Studer et al., 1994; Popperl et al., 1995), and by persistent expression of *Hoxb2* (Barrow and Capecchi, 1996; Gavalas et al., 2003; Pattyn et al., 2003a; Di Bonito et al., 2013a), whereas *Hoxa1* is downregulated (Murphy and Hill, 1991). *Hoxa2* and *Hoxb2* are members of the PG2 *Hox* genes expressed with an anterior border at the r1/r2 and r2/r3 boundaries, respectively (Wilkinson et al., 1989; Hunt et al., 1991; Krumlauf, 1993a; Prince and Lumsden, 1994). *Hoxa2* is expressed at low levels in r2 and r4 and at high levels in r3, particularly in a wide intermediate dorsal column and in a thinner more lateral one, the presumptive auditory column (Di Bonito et al., 2013a). *Hoxb2* is co-expressed with *Hoxa2* in r3 at low levels and with *Hoxb1* in r4 at high levels (Di Bonito et al., 2013a). *Hoxb1* upregulates *Hoxb2* in r4 by binding to an r4-specific enhancer at the 5′ region of the *Hoxb2* locus (Maconochie et al., 1997; Ferretti et al., 2000) and downregulates *Hoxa2* (Di Bonito et al., 2013a), whereas *Krox20* is an upstream regulator of *Hoxa2* (Nonchev et al., 1996) and *Hoxb2* (Sham et al., 1993) in r3 and r5. In the auditory system, *Hoxb2* and *Hoxa2* are still maintained in the CN, VLL, and SOC nuclei during prenatal and postnatal developmental stages (Narita and Rijli, 2009; Di Bonito et al., 2013a). Within the CN, *Hoxa2* is mainly expressed in the AVCN, which originates from r2 and r3, whereas *Hoxb2* is expressed in r4-derived structures, such as the PVCN, and in r3-derived AVCN and cochlear granule cells. Moreover, both genes are maintained in the r3/r4/r5 derived DCN (Farago et al., 2006; Di Bonito et al., 2013a).

Below, we will go into more details in the function of those *Hox* genes directly involved in the formation of the auditory system.

### *Hoxa1* acts as a strong selector gene of r4 and r5 during hindbrain patterning

Deletion of *Hoxa1* leads to severe early defects in the hindbrain, since *Hoxa1*^*null*^ embryos have a reduced r4 and almost a complete loss of r5 structures (Carpenter et al., 1993; Dolle et al., 1993; Mark et al., 1993). In the absence of these structures and their neural crest derivatives, *null* mice completely lack or have severe defects in the formation of middle ear ossicles (Lufkin et al., 1991; Chisaka et al., 1992; Mark et al., 1993), of the auricle and of the external acoustic meatus (Chisaka et al., 1992). The SG, the cochlear component of the VIIIth vestibulocochlear ganglion, is also lost. In the hindbrain, FBM and SOC nuclei are absent or strongly reduced, in agreement with reduction of r4 and almost complete loss of r5, whereas the VCN and DCN appear quite normal (Lufkin et al., 1991; Chisaka et al., 1992; Mark et al., 1993).

### *Hoxb1* acts as a determinant gene of r4 sensory identity and r4-derived auditory neurons

Differently from *Hoxa1, Hoxb1* acts as a true homeotic gene by imprinting a r4 identity in the developing hindbrain. Since r4 contributes to several auditory structures, as described above, *Hoxb1* represents a key developmental gene playing a crucial role in the establishment of regional identity of motor (Studer et al., 1996; Gaufo et al., 2000; Gavalas et al., 2003; Pattyn et al., 2003a; Di Bonito et al., 2013a) and sensory r4-derived structures (Gaufo et al., 2004; Chen et al., 2012; Di Bonito et al., 2013a, 2015), partly by promoting *Hoxb2* and repressing *Hoxa2* expression in r4 (Maconochie et al., 1997; Ferretti et al., 2000; Tumpel et al., 2007; Di Bonito et al., 2013a). In *Hoxb1* mutants, in which *Hoxb1* is either constitutive (*Hoxb1*^*null*^) or conditionally (*Hoxb1*^*lateCKO*^) inactivated in r4, *Hoxb2* is reduced and *Hoxa2* is increased at expression levels similar to those in r3 (Di Bonito et al., 2013a). This is also observed in *Hoxb2*^Δ*KO*^ embryos, in which *Hoxb1* fails to be maintained in r4. The re-patterning of r4 into an r3-like identity, as a result of *Hoxb1* inactivation, ultimately leads to the loss of specific r4-derived auditory nuclei (FBM, MOC, LOC, VLL, and PVCN) and to the ectopic formation and functional acquisition of neuronal populations that normally derive from r3 (TBM, AVCN and cochlear granule cells) (Studer et al., 1996; Di Bonito et al., 2013a). *Hoxb1*^*lateCKO*^ and *Hoxb2*^Δ*KO*^ mutants exhibit a similar though milder phenotype than *Hoxb1*^*null*^ mice, since early *Hoxb1* expression is able to partially specify r4 identity in these mutants, but failure to maintain *Hoxb1* at later stages inhibits further development of r4-derived auditory nuclei (Di Bonito et al., 2013a). This concerns mainly the r4-derived nuclei that are developing while *Hoxb1* is inactivated (FBM, OC, and VLL), whereas nuclei generated after *Hoxb1* inactivation, such as the CN, have similar defects in all mutants.

**Figure 8:**
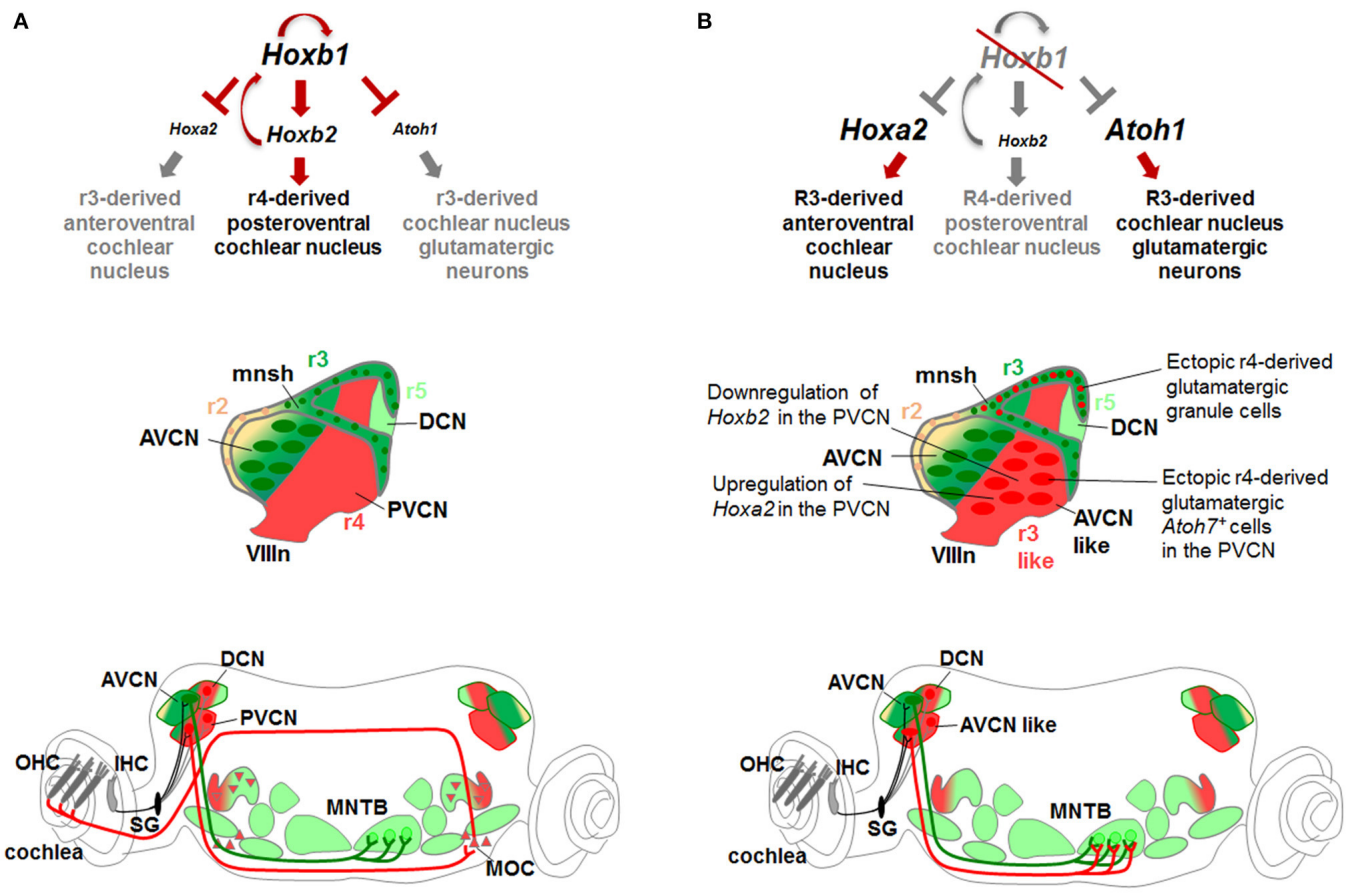
***Hox* gene networks involved in the assembly of central auditory subcircuits. (A)** In r4, *Hoxb1* activates *Hoxb2* and represses *Hoxa2* and *Atoh1* preventing the formation of *Atoh1-*derived glutamatergic neurons normally produced in r3. **(B)** In the absence of *Hoxb1, Atoh1* and *Hoxa2* are upregulated while *Hoxb2* is downregulated at similar levels than in r3. Ectopic r4-derived granule cells contribute to the microneuronal shell. The r4-derived PVCN acquires “AVCN-like” identity producing ectopic glutamatergic *Atoh7*^+^ spherical bushy cells and projecting to the MNTB, a physiologically target of r3-derived AVCN neurons. Modified from Di Bonito et al. (2013a).

*Hoxb1* is an important determinant gene in the r4-derived CN development in part by inhibiting a r3-like genetic pathway. One of the key *Hox* genes involved in patterning the AVCN in r3 is *Hoxa2*, which is instead expressed at low levels in r4 differentiating cells, playing thus only a minor role in patterning r4-derived CN (Di Bonito et al., 2013a). In the absence of *Hoxb1, Hoxa2* expression in r4 is increased at similar levels to r3, whereas *Hoxb2* is downregulated (Di Bonito et al., 2013a; Figure 8). *Hoxa2* and *Atoh7*, normally highly expressed in r3-derived AVCN neurons, are now ectopically upregulated in the r4-derived PVCN of *Hoxb1*^*null*^ mutants leading ultimately to a change of PVCN to AVCN identity and abnormal connectivity pattern. Ectopic *Atoh7*^+^ glutamatergic neurons abnormally differentiate in the mutant PVCN acquiring the fate of spherical bushy cells (Di Bonito et al., 2013a), which are normally r3-derived AVCN neurons produced by the *Atoh1* domain (Farago et al., 2006; Saul et al., 2008; Fujiyama et al., 2009; Maricich et al., 2009). As a consequence, the AVCN-like mutant neurons of PVCN abnormally project to the MNTB, the physiological target of spherical bushy cells (Saul et al., 2008; Di Bonito et al., 2013a). Moreover, r4-derived mutant cells acquire the identity of granule cells, which normally do not originate from r4 but from the *Atoh1*^+^ domain in r2/r3, and massively invade the glutamatergic microneuronal shell, which maintains *Hoxb2* expression. The ectopic production of glutamatergic granule cells and of *Atoh7*^+^ neurons in the mutant r4-derived CN seems to be correlated with increased *Atoh1* expression observed at early stages. This is in accordance with the fact that *Atoh1* regulates *Atoh7* expression levels, and that granule cells and *Atoh7*^+^ neurons are both glutamatergic *Atoh1*-derivatives (Hufnagel et al., 2007). It is thus plausible that *Hoxb1* normally inhibits *Atoh1* in r4, since the change of r4 to r3 identity as a result of *Hoxb1* inactivation, leads to an enlargement of the r4 *Atoh1*^+^ sensory domain (normally smaller in r4 than in adjacent rhombomeres) and to ectopic production of cochlear *Atoh1*-derived excitatory populations (such as cochlear granule cells and *Atoh7*^+^ neurons) (Di Bonito et al., 2013a). Overall, *Hoxb1* negatively modulates *Hoxa2* and *Atoh1* during r4 patterning and thus inhibits the specification of r3-derived CN structures (Figure 8).

As previously mentioned, *Ptf1a* is required for the generation of inhibitory GABAergic and glycinergic neurons in the CN and cerebellum, whereas excitatory glutamatergic neurons derive from the *Atoh1-*lineage (Hoshino et al., 2005; Fujiyama et al., 2009; Yamada et al., 2014). Moreover, *Ptf1a* determines GABAergic over glutamatergic neuronal cell fate in the cerebellum and spinal cord (Glasgow et al., 2005; Pascual et al., 2007). In the absence of *Ptf1a*, neurons derived from this lineage abnormally express *Atoh1* and ectopically generate *Atoh1*-derived glutamatergic granule cells in the cerebellum (Pascual et al., 2007). Based on this evidence, it is plausible that altered *Ptf1a* expression might be responsible for the ectopic *Atoh1* expression in dorsal r4 of *Hoxb1* mutant mice. Thus, *Ptf1a* may act downstream of *Hoxb1* to inhibit *Atoh1* and the r3-like glutamatergic fate of r4-derived cochlear neurons. Alternatively, *Hoxb1* might regulate both *Ptf1a* and *Atoh1* expression levels in sensory r4. Intersectional long-term fate mapping using an r4-*Flp* line and neuronal DV subtype *Cre*-specific lines will be crucial to fully characterize individual r4 subpopulations and elucidate this aspect, as well as determine the unknown contribution of the *Atoh1*^+^ domain in r4.

*Hoxb1* is also an important determinant gene in the r4-derived VLL development. Fate mapping analysis showed that the VLL originates from r4 through rostral migration. Upon loss of *Hoxb1* at early stages, the majority of r4 VLL neurons are not specified leading to a strongly reduced *Gad67*^+^ GABAergic/glycinergic VLL nucleus expressing *Gata3*. Notably, loss of *Hoxb1* at later stages (in *Hoxb1*^*lateCKO*^ or *Hoxb2*^Δ*KO*^ mutant mice) results in a less severe reduction of the VLL (Di Bonito et al., 2013a), suggesting that VLL cells start to be patterned before E9.0. Finally, *VGluT2*-expressing neurons in the LSO of *Hoxb1*^*null*^ are reduced but not completely lacking, in agreement with the evidence that glutamatergic LSO neurons derive not only from r4 (Di Bonito et al., 2013a), but have also an additional contribution from r5 (Maricich et al., 2009; Marrs et al., 2013; Altieri et al., 2015).

### *Hoxb1* acts as a determinant gene of ventral r4-derived motor neurons

Regarding the ventral r4 domain, two pools of visceral motor neurons (MNv), FBM and IEE (Simon and Lumsden, 1993; Auclair et al., 1996; Bruce et al., 1997), originate from a common ventral progenitor domain expressing *Nkx2.2* (pMNv) (Briscoe et al., 2000; Pattyn et al., 2003a,b) (Figure 5B). During differentiation, however, the two populations display distinct migratory behaviors. While FBM neurons migrate caudally from r4 to r6 where they form the facial nucleus of the VIIth cranial nerve (Auclair et al., 1996; Studer et al., 1996; Bruce et al., 1997), IEE neurons project contralaterally through the midline in r4 by somatic translocation before splitting and forming the VEN (dorsal and lateral to the internal genu of the facial nerve) and the CEN, which will give rise to the LOC and MOC neurons (respectively within and at the margin of LSO and in the medioventral part of SOC) (Simon and Lumsden, 1993; Bruce et al., 1997; Simmons, 2002; Tiveron et al., 2003; Brown and Levine, 2008; Figures 4A,C, 5A,D). *Hoxb1* is required for the specification of IEE and FBM motor neurons, as demonstrated by loss- and gain-of-function studies (Goddard et al., 1996; Studer et al., 1996; Bell et al., 1999; Jungbluth et al., 1999). *Hoxb1*^*null*^ embryos fail to specify both FBM and IEE (Goddard et al., 1996; Studer et al., 1996) and r4 motor neurons migrate dorsally acquiring a r3-derived trigeminal neuron-like migratory behavior (Studer et al., 1996). In *Hoxb1*^*lateCKO*^ and in *Hoxb2*^Δ*KO*^ embryos, r4 ventral neurons acquire a mixed identity, whereby a portion migrate caudally following the normal migratory path of FBM (and reaching an ectopic final position in r5 in *Hoxb1*^*lateCKO*^), while a second population follow a trigeminal-like pattern of migration (Gavalas et al., 2003; Di Bonito et al., 2013a, and unpublished data). IEE-derived efferent *ChAT*- and *Tbx20*-expressing MOC and LOC neurons are lost in *Hoxb1*^*null*^ mice, whereas some LOC neurons and few MOC connections are partially specified in *Hoxb1 conditional* mutants (Di Bonito et al., 2013a), in agreement with the timing of *Hoxb1* gene inactivation. On the contrary, *Hoxb1* global overexpression (Bell et al., 1999; Pata et al., 1999) or ectopic expression in r1 (Jungbluth et al., 1999) results in misspecification of neurons with identity and axonal pathfinding characteristic of r4 efferent neurons. This confirms that *Hoxb1* is necessary and sufficient to impart an r4 motor identity in the developing hindbrain.

Besides *Nkx2.2*, the pMNv also expresses other transcription factors such as *Nkx2.9* (Briscoe et al., 1999), *Nkx6.1* and *Nkx6.2* (Briscoe et al., 2000), *Phox2b, Mash1* (Pattyn et al., 2000), and *Gata2* (Nardelli et al., 1999; Pata et al., 1999; Tiveron et al., 2003) (Figure 5B). This domain normally produces at early stages MNv and at late stages serotonergic neurons along the developing hindbrain, but not in r4 where pMNv progenitors continue to form motor neurons. Intrinsic to its role as an r4 identity gene, *Hoxb1* promotes MNv and suppresses serotonergic neuronal fate by extending the spatial and temporal activation of *Phox2b* expression in *Nkx2.2*^+^ progenitors (Pattyn et al., 2003a; Jacob et al., 2007). *Hoxb1* maintains *Phox2b* expression through direct positive regulation (Gaufo et al., 2000; Samad et al., 2004), while *Phox2b* inhibits *Foxa2* and the serotonergic fate during sustained MNv neurogenesis in r4 (Jacob et al., 2007). Loss of MNv neurons and ectopic production of serotonergic neurons is therefore observed in r4 of both *Hoxb1*^*null*^ and *Phox2b*^*null*^ mutant mice in which *Foxa2* expression expands dorsally into the *Nkx2.2*^+^ pMNv domain (Jacob et al., 2007). The initial phase of *Hoxb1* expression in the *Nkx2.2*^+^ domain in r4 is unaffected in *Nkx6* (*Nkx6.1* and *Nkx6.2* compound) and in *Hoxb2* mutant mice until E10.5 and E11.5, respectively. Consequently, MNv generated before *Hoxb1* downregulation retain r4 characteristics, while late-born neurons adopt a serotonergic neuronal fate, demonstrating that *Hoxb1* expression is required over time allowing progenitors to maintain r4-positional identity and to acquire their appropriate neuronal fate (Pattyn et al., 2003a).

Besides regulating *Phox2b* in r4 pMNv progenitors, *Hoxb1* also induces the zinc finger transcription factor *Gata2*, which in turn activates its homolog *Gata3* (Nardelli et al., 1999; Pata et al., 1999). Both are expressed at high levels in IEE neurons but not in FBM postmitotic neurons (Pata et al., 1999; Karis et al., 2001). By E11.5, *Gata2* expression is downregulated in ventral r4, which continues to express *Gata3* (Nardelli et al., 1999; Pata et al., 1999). Absence of *Hoxb1* results in the loss of both *Gata* gene expression in r4 and, conversely, ubiquitous expression of *Hoxb1* in the hindbrain induces ectopic expression of *Gata2* and *Gata3* (Nardelli et al., 1999; Pata et al., 1999; Karis et al., 2001). *Phox2b*, downstream of *Hoxb1* in r4, is also required in maintaining high *Gata3* expression levels in IEE neurons, since low *Gata3* expression is required for serotonergic differentiation. In *Phox2b*^*null*^ embryos, IEE fail to differentiate and weak *Gata3* expression is maintained in ventral serotonergic precursors throughout the length of the hindbrain including r4 (Tiveron et al., 2003). In *Gata3*^*null*^ embryos, migration of IEE appears to happen normally, but IEE projections are reduced with only few fibers reaching the contralateral ear. In addition, the majority of OC and vestibular efferent fibers rearrange their trajectory and project along the facial and greater petrosal nerves bypassing the ear, supporting a role for Gata3 in IEE axonal pathfinding (Karis et al., 2001; Duncan et al., 2011). In summary, *Hoxb1* promotes generation of ventral motor neurons by maintaining high expression levels of *Phox2b* (for FBM and IEE) and *Gata3* (for IEE) in ventral r4 and repressing thus a r3-like serotonergic fate.

### Interactions between efferent neurons and hair cells during cochlear functional maturation

In the absence or aberrant differentiation of IEE, as observed in *Hoxb1* mutant embryos, OHCs degenerate mainly in the apical cochlear portion, causing the altered hearing threshold described in adult mice (Di Bonito et al., 2013a). In particular, the transmission of low-level auditory stimuli is strongly affected resulting in severe hearing impairments. One hypothesis is that OHC degeneration might be caused by the absence of synaptic/trophic innervation from efferent OC fibers during a postnatal critical period, shown to be important for proper OHC maturation (Walsh et al., 1998; Di Bonito et al., 2013a). This is mainly supported by the observation that in *Hoxb1*^*null*^ mutants OHC morphology is not affected until early postnatal stages, but impaired only after synaptic MOC/OHC interactions normally occur, which instead fail to be formed in the absence of MOC neurons. Moreover, the persistence of some MOC neurons innervating OHCs in *Hoxb1*^*lateCKO*^ and *Hoxb2*^Δ*KO*^ mutants, in which *Hoxb1* is not fully abolished, correlates with milder OHC morphological and hearing threshold defects (Di Bonito et al., 2013a). The importance of early interactions between efferent neurons and their postsynaptic targets for proper cochlear functional maturation had been previously suggested by deefferentation studies in the cats. Surgical physical deefferentation in neonatal cats, but not in adult cats, resulted in threshold elevations and other cochlear response abnormalities consistent with OHC dysfunctions (Walsh et al., 1998). However, in the absence of OHC nicotinic acetylcholine receptor subunits α9 (Vetter et al., 1999; Brown and Vetter, 2009) or α10 (Vetter et al., 2007), the MOC/OHC efferent innervation is still preserved even if terminals contacting OHC are abnormal in size and numbers. Surprisingly, even if the MOC inhibition on OHC activity cannot be transduced (due to the lack of these receptors) leading thus to a complete “functional deefferentation,” the cochlear response is maintained with no abnormalities in ABR thresholds or DPOAE amplitudes (Vetter et al., 1999, 2007). This is different from the surgical physical deefferentation described above, since OHCs seem to function normally despite the inability of OHCs to respond to the MOC inhibition. However, in these mutants where the MOC innervation is still maintained, only one of the two α-nicotinic receptor subunits is eliminated, while several other synaptic proteins are preserved at the MOC/OHC synapse, whose contribution could compensate. It is thus premature to conclude that the lack of a significant developmental effect and the apparent normal cochlear responses in the acetylcholine receptors mutant mice contradict the influence of efferent terminals on the functional OHC maturation. Alternatively, the action of the efferent system on the functional development of OHCs might be mediated by a process not involving α9- and α10-nicotinic receptors (Vetter et al., 1999; Simmons, 2002). Future genetic, such as double α*9/*α*10 KOs*, and sophisticated functional studies might further clarify this issue.

Altered development of auditory sensory structures might also contribute to the hearing phenotype of *Hoxb1*^*null*^ mice. Even if this possibility cannot be completely ruled out, the persistence of few MOC neurons in some *Hoxb1*^*lateCKO*^ mutant mice correlates well with less severe OHC morphology and threshold impairments, although other auditory sensory nuclei are similarly affected in all *conditional* mutants (Di Bonito et al., 2013a and unpublished data). This strongly suggests that early interactions between OC and OHC might be crucial for proper maturation of hair cells. Thus, to ultimately discern between these possibilities, *Hoxb1* should be inactivated either in the sensory or only in the motor r4-derived components of the auditory pathway. Finally, the progressively more drastic increase of auditory threshold with age observed in *Hoxb1* adult mutant mice is likely due to the reduced function of efferent (MOC and MEM) reflexes, as well as LOC neurons, which cannot protect the organ of Corti from noise-induced hearing damages. Accordingly, *Hoxb1 conditional* mice, whose efferent neurons (MOC, LOC, and FBM) are partially preserved have a less severe increase of auditory threshold with age compared to *Hoxb1*^*null*^ mice in which all efferent motor neurons are completely absent (Di Bonito et al., 2013a).

A similar hearing loss phenotype due to degeneration of OHCs with consequent increase of auditory threshold has also been described in heterozygous *Gata3* mutant mice, a model of *GATA3* haploinsufficiency that causes the human autosomal dominant HDR (Hypoparathyroidism, Deafness and Renal dysplasia) syndrome (Bilous et al., 1992; Van Esch et al., 2000; van der Wees et al., 2004). In particular, the hearing threshold elevation of 30 dB and reduction of OHC functionality in heterozygous *Gata3* mice seem to be caused by the progressive morphological degeneration of OHCs at the apex followed by progressive degeneration of all hair cells and supporting cells in the entire cochlea (van der Wees et al., 2004; van Looij et al., 2005). *Gata3* is continuously expressed from early development to adulthood in different parts of both the inner ear and the central auditory nervous system. Indeed, *Gata3* is expressed in the SG neurons and in virtually all cell types of the inner ear including IHCs, OHCs and supporting cells (Karis et al., 2001; Lawoko-Kerali et al., 2002) and plays a crucial role in the inner ear neurosensory cochlear development, as demonstrated by *constitutive* and *conditional KO* studies (Haugas et al., 2012; Appler et al., 2013; Duncan and Fritzsch, 2013; Luo et al., 2013). In the central auditory system, *Gata3* is found in the SPO, VLL, IC, and IEE efferent neurons (Karis et al., 2001; van der Wees et al., 2004; Di Bonito et al., 2013a). Since it is also expressed in the OHCs, it is highly probable that reduced levels of *Gata3* within the cochlea might be the major cause of hearing impairments. However, *Gata3* is also involved in driving IEE fibers to the ear. Thus, it is also plausible that absence of innervation of OHCs by OC neurons might contribute to the hearing loss caused by *GATA3* haploinsufficiency in the human HDR syndrome. *Conditional* and intersectional deletion studies inactivating *Gata3* either in the CNS or in the inner ear will be useful to dissect the central and peripheral functional roles of *Gata3* during formation of the auditory system.

### *Hoxb2* expression is maintained in r3- to r5-derived auditory nuclei

*Hoxb2* acts in maintaining high expression of *Hoxb1* in r4 progenitors since *Hoxb1* expression is initiated but not maintained in r4 of *Hoxb2* mutants (Barrow and Capecchi, 1996; Gavalas et al., 2003; Pattyn et al., 2003a; Di Bonito et al., 2013a) and *Hoxb2*^Δ*KO*^ mice reproduce similar phenotypes as *Hoxb1*^*lateCKO*^ mice, in which only late expression of *Hoxb1* is eliminated (Di Bonito et al., 2013a). Moreover, *Hoxb2* is a direct target activated by *Hoxb1* in r4 progenitors (Maconochie et al., 1997; Ferretti et al., 2000) and, importantly, is maintained in r4-derived auditory nuclei, such as the VLL and PVCN (Narita and Rijli, 2009; Di Bonito et al., 2013a), suggesting that *Hoxb2* plays a role in relaying r4-dependent regional fate to postmitotic neurons. Moreover, *Hoxb2* is also expressed in r3 and r5 together with *Hoxa2* and maintained in r3-derived granule cells and AVCN (Di Bonito et al., 2013a) and in r5-derived SOC nuclei (Narita and Rijli, 2009). A transgenic line activating the *Cre recombinase* under the control of a *Hoxb2* r4 enhancer crossed with a *R26R LacZ* reporter shows labeling in the early otocyst, in the vestibular and spiral ganglia, and in all otic epithelia at later stages (Szeto et al., 2009). Thus *conditional* inactivation of *Hoxb2* after E13.5, when *Hoxb1* expression is downregulated in r4, or specifically in r3 and/or r5 would help in unraveling *Hoxb2* specific roles in auditory central nuclei. In addition, the use of the *b2-r4-Cre* transgenic mice (Szeto et al., 2009) might provide an important tool for *conditional* gene ablation and lineage tracing in the inner ear.

### *Hoxa2* is an important determinant gene of r2/r3-derived anterior cochlear nucleus

Inactivation of *Hoxa2* results in lethality at birth and in a homeotic transformation of structures derived from the 2nd arch neural crest into 1st arch derivatives (Gendron-Maguire et al., 1993; Rijli et al., 1993). As a result, *Hoxa2*^*null*^ mutants exhibit defects in the formation of the middle and external ears: in the middle ear, the stapes and stapedius muscles (2nd pharyngeal arch neural crest derivatives) are lost with a duplication of the malleus and incus primordia and enlargement of the muscles of the malleus, the tensor tympani (1st pharyngeal arc neural crest derivatives). In the hindbrain, *Hoxa2*, strongly expressed in r3 and at lower levels in r2, plays a key role in the segmental identity of these rhombomeres and is required in patterning the r2/r3-derived AVCN (Gavalas et al., 1997; Farago et al., 2006). Loss of *Hoxa2* mainly impairs the development of the AVCN, which is drastically reduced in *Hoxa2*^*null*^ mice, and the respective projections to the contralateral MNTB nucleus as described in *Hoxa2 conditional* mutant mice (Gavalas et al., 1997; Di Bonito et al., 2013a). In the AVCN, *Hoxa2* controls expression of the *Slit* receptor *Rig1/Robo3* (Di Bonito et al., 2013a), known to regulate midline crossing of commissural axons in the hindbrain (Renier et al., 2010). Early *conditional Hoxa2* inactivation in rhombic-lip derivatives selectively affects *Rig1* expression and AVCN axonal pathfinding to MNTB, resulting in reduced contralateral and ectopic ipsilateral fibers targeting the MNTB (Di Bonito et al., 2013a). It is unlikely that the *Hoxa2*-mediated regulation of *Rig1* alone could explain the PVCN-to-AVCN target connectivity switch observed in *Hoxb1* and *Hoxb2* mutant mice, since Rig1 only confers the ability of axons to cross the midline. Most probably, *Hoxa2* acts as an important determinant of a larger transcriptional program required to specify r2/r3-derived AVCN neurons with their proper identity and connectivity. Even if the domain of *Atoh7* expression is reduced in the smaller AVCN of *Hoxa2*^*null*^ embryos, *Atoh7* continues to be expressed in the residual CN cells indicating that *Atoh7* is not under the transcriptional control of *Hoxa2* (Di Bonito et al., 2013a). *Hoxa2* and *Atoh7*, which is downstream of *Atoh1*, could be part of two parallel pathways essential for AVCN formation, identity and connectivity. Both pathways may be specific for r3 and normally downregulated in r4 by *Hoxb1*-mediated inhibition of *Hoxa2* and *Atoh1* (Figure 8). A new role of *Hoxa2* in the AVCN has been recently reported. *Hox2* genes are required for the proper innervation of *Atoh1*-derived glutamatergic bushy cells by SG afferent neurons and sound-frequency discrimination, indicating a role for these *Hox* factors in tonotopic refinement of connectivity and in ensuring the precision of sound transmission in the mammalian auditory circuit (Karmakar et al., 2017).

Differently from the AVCN, *Hoxa2* do not appear to play a predominant role in VLL specification since no size abnormalities have been observed in the VLL during development (Di Bonito et al., 2013a). However, we cannot exclude that *Hoxa2* might be involved in axonal pathfinding of VLL neurons similar to what described for the AVCN (Di Bonito et al., 2013a) and for the principal trigeminal sensory PrV (Oury et al., 2006) nuclei. The role of *Hoxa2* in the SOC remains to be elucidated.

## Conclusions and perspectives

Thanks to the availability of several mouse transgenic lines, significant progress in the identification and formation of auditory sensorimotor hindbrain subcircuits has been obtained in the last few years. It is now well accepted in the field that rostral rhombomeres and their associated *Hox* genes are involved in establishing and maintaining two major functional circuits in the central auditory system. *Hoxb1* and *Hoxb2* appear to act primarily in the assembly of r4-derived structures by contributing to the main sound transmission pathway, as well as in the establishment of sensorimotor reflex circuits important for the cochlear amplification and protection (Figure 6). *Hoxa2* instead seems to contribute to AVCN development and connectivity in the sound localization circuitry mainly formed by r2, r3, and r5 (Figure 7).

Despite the considerable progress made in the field, we still need to understand the exact origin, cellular behavior (tangential vs. radial migration), connectivity patterns and molecular profile of several auditory neuronal populations. The intersectional strategy using both *Cre-* and *Flp-recombinase* mouse lines together with a dual reporter transgenic line will increase the opportunity to label, mutate and isolate restricted hindbrain subpopulations, and thus represents an important tool for future studies (Dymecki et al., 2010). By intersectional lineage tracing, distinct rhombomere-specific DV subdomains contributing to several auditory nuclei and subcircuits can be identified and labeled, and single neuronal populations can be traced from embryonic stages to adulthood. The comparison of mutant backgrounds will unravel how different subdomains and neuronal derivatives contribute to the normal and diseased acoustic pathways and allow identification of their functional role. Moreover, the intersectional fate mapping will be helpful in dissecting rhombomere-specific molecular auditory pathways of single DV subdomains and deciphering how correspondent domains of different rhombomeres can contribute to distinct auditory neurons with different functions. In mouse models of hearing impairments, the intersectional strategy could be used to isolate selected neuronal subpopulations and identify, through genomic approaches, novel molecular players that are involved in the specification and circuit assembly of auditory neuronal subtypes, and eventually affected in hearing loss pathologies of mice and humans. The identification of the molecular, anatomical and functional properties of distinct auditory neuronal populations in normal and pathological conditions will help in unraveling the genetic causes of deafness and characterizing novel genes involved in hearing. This will allow designing novel molecular genetic tests for hearing diagnosis and genetic counseling, and provide basic knowledge for clinical research of future therapies.

## Author contributions

All authors listed, have made substantial, direct and intellectual contribution to the work, and approved it for publication.

### Conflict of interest statement

The authors declare that the research was conducted in the absence of any commercial or financial relationships that could be construed as a potential conflict of interest.

## Abbreviations

V: Trigeminal motor nucleus
VII: Facial motor nucleus
VIII: Vestibulocochlear nuclei
VIIIn: VIIIth cranial nerve
X: Dorsal vagal motor nucleus
XII: Hypoglossal motor nucleus
Amb: Ambiguus motor nucleus
AP: Area postrema
AVCN: Anteroventral cochlear nucleus
Cb: Cerebellum
CES: Cochlear extramural stream
CN: Cochlear Nucleus
Cx: Cerebral cortex
DAS: Dorsal acoustic stria
DCN: Dorsal cochlear nucleus
dcn: Dorsal column nuclei
DLL: Dorsal nucleus of the lateral lemniscus
ECn: External cuneate nucleus
FBM: Facial branchiomotor neuron
FL: Floor plate
GBC: Globular bushy cell
IAS: Intermediate acoustic stria
IC: Inferior colliculus
IHC: Inner hair cell
ILL: Intermediate nucleus of the lateral lemniscus
LOC: Lateral olivocochlear neurons
LNTB: Lateral nucleus of the trapezoid body
LRL: Lower rhombic lip
LSO: Lateral superior olive
MG: Medial geniculate nucleus
MOC: Medial olivocochlear neurons
MNs: Somatic motor neuron
mnsh: Microneuronal shell
MNTB: Medial nucleus of trapezoid body
MNv: Visceral motor neurons
MSO: Medial superior olive
MZ: Mantle zone
NC: Notochord
OHC: Outer hair cell
PrV: Principal trigeminal nucleus
PVCN: Posteroventral cochlear nucleus
RAmb: Retroambiguus nucleus
RP: Roof plate
SBC: Spherical bushy cell
SC: Spinal cord
SG: Spiral ganglion
TBM: Trigeminal branchiomotor neuron
SOC: Superior olivary complex
SPO: Superior paraolivary nucleus
TB: Trapezoid body
VCN: Ventral cochlear nucleus
VLL: Ventral nucleus of the lateral lemniscus
VNTB: Ventral nucleus of the trapezoid body
VZ: Ventricular zone.
